# A scoping review on bovine tuberculosis highlights the need for novel data streams and analytical approaches to curb zoonotic diseases

**DOI:** 10.1186/s13567-024-01314-w

**Published:** 2024-05-21

**Authors:** Kimberly Conteddu, Holly M. English, Andrew W. Byrne, Bawan Amin, Laura L. Griffin, Prabhleen Kaur, Virginia Morera-Pujol, Kilian J. Murphy, Michael Salter-Townshend, Adam F. Smith, Simone Ciuti

**Affiliations:** 1 Laboratory of Wildlife Ecology and Behaviour, School of Biology and Environmental Science, University College Dublin, Dublin, Ireland; 2 Department of Agriculture, Food and the Marine, One Health Scientific Support Unit, Dublin, Ireland; 3 School of Mathematics and Statistics, University College Dublin, Dublin, Ireland; 4 Department of Wildlife Ecology and Management, Faculty of Environment and Natural Resources, University of Freiburg, Freiburg, Germany; 5 The Frankfurt Zoological Society, Frankfurt, Germany; 6 Department of National Park Monitoring and Animal Management, Bavarian Forest National Park, Grafenau, Germany

**Keywords:** zoonosis, bovine tuberculosis, *Mycobacterium bovis*, infectious disease management, mathematical modelling, simulation, multi-host disease, wildlife host

## Abstract

Zoonotic diseases represent a significant societal challenge in terms of their health and economic impacts. One Health approaches to managing zoonotic diseases are becoming more prevalent, but require novel thinking, tools and cross-disciplinary collaboration. Bovine tuberculosis (bTB) is one example of a costly One Health challenge with a complex epidemiology involving humans, domestic animals, wildlife and environmental factors, which require sophisticated collaborative approaches. We undertook a scoping review of multi-host bTB epidemiology to identify trends in species publication focus, methodologies, and One Health approaches. We aimed to identify knowledge gaps where novel research could provide insights to inform control policy, for bTB and other zoonoses. The review included 532 articles. We found different levels of research attention across episystems, with a significant proportion of the literature focusing on the badger-cattle-TB episystem, with far less attention given to tropical multi-host episystems. We found a limited number of studies focusing on management solutions and their efficacy, with very few studies looking at modelling exit strategies. Only a small number of studies looked at the effect of human disturbances on the spread of bTB involving wildlife hosts. Most of the studies we reviewed focused on the effect of badger vaccination and culling on bTB dynamics with few looking at how roads, human perturbations and habitat change may affect wildlife movement and disease spread. Finally, we observed a lack of studies considering the effect of weather variables on bTB spread, which is particularly relevant when studying zoonoses under climate change scenarios. Significant technological and methodological advances have been applied to bTB episystems, providing explicit insights into its spread and maintenance across populations. We identified a prominent bias towards certain species and locations. Generating more high-quality empirical data on wildlife host distribution and abundance, high-resolution individual behaviours and greater use of mathematical models and simulations are key areas for future research. Integrating data sources across disciplines, and a “virtuous cycle” of well-designed empirical data collection linked with mathematical and simulation modelling could provide additional gains for policy-makers and managers, enabling optimised bTB management with broader insights for other zoonoses.

**Supplementary Information:**

The online version contains supplementary material available at 10.1186/s13567-024-01314-w.

## Introduction

Emerging infectious diseases represent a significant public health concern as they become more prevalent worldwide [1–3]. It is estimated that about 60% of emerging infectious diseases are zoonotic, 72% of which have been estimated to originate from wildlife [2, 3]. In 2019, thirteen different zoonoses had confirmed cases in humans within the European Union [4]. This has likely been accelerated by exponential growth in global population size and mobility with associated increases in urbanisation and concurrent loss of natural habitats. It has also led to increasing occurrences of human-wildlife interactions (e.g., improper waste disposal, intentional feeding of wildlife, movement of wildlife to human-dominated areas) and, therefore, exposure to zoonotic diseases [5–7]. Contact between humans, livestock and other captive animals, and wildlife species is only expected to keep increasing, leading to concerns about increased incidences of zoonotic disease transfer [6, 8, 9]. The question, however, remains of how to best track and manage emerging diseases.

A critical example is Zoonotic Tuberculosis (zoonotic TB), which was estimated in 2016 to be linked to 147 000 human cases and 12 500 deaths worldwide [10]. Zoonotic TB is driven mainly by *Mycobacterium bovis* (i.e., the causative agent of Bovine Tuberculosis—also as bovine TB or bTB), which is transmitted by several wildlife hosts and livestock. Britain and Ireland, as well as many other countries worldwide [10], have been increasingly impacted by bTB, resulting in significant economic loss. In Ireland, for instance, 4.89% of cattle herds tested positive for bTB in 2023, leading to the humane killing of 28 868 cattle [11]. This is in addition to the economic costs associated with the national bTB eradication program with €92 million spent in 2018 alone [12]. Similar trends can be observed in the UK, with £70 million spent annually for bTB prevention and control [13]. This disease also raises welfare concerns for wildlife hosts, especially considering its high prevalence in the wild. Badgers (*Meles meles*), for example, have been shown to have a bTB prevalence exceeding 40% in hotspot areas in Ireland [14], and red deer in Spain have been estimated to have a prevalence of up to 50% [15].

Bovine TB eradication is prioritised by governments and researchers due to the significant health concerns and economic (trade) impacts. Despite decades of control efforts in several countries, the pathogen has successfully avoided eradication. There are complex reasons as to why this is the case [16], but a primary factor relates to its complex dynamics of transmission and maintenance across differing hosts and the environment. Therefore, new thinking may be required to further investigate if disease control can be driven toward eradication. Detecting gaps in the current bTB literature is an essential step required to identify target areas for future research and to further hone government eradication strategies.

One way in which this may be addressed, and which requires assessment as to its prevalence in the literature, is through multidisciplinary, coordinated collaborations between the public health sector, veterinarians, ecologists and wildlife managers. The importance of interdisciplinary approaches is highlighted by the interlinked nature of human, animal and ecosystem health, which led to the concept of “One World One Health™” [17, 18]. Despite such multidisciplinary efforts, the effect of stressors (i.e., direct and/or indirect disturbances such as hunting, habitat loss, and more broadly habitat and climate change) on animal ecology within human-dominated landscapes and the potential emergence of zoonotic disease is still understudied [1]. For example, we are aware that human-driven changes in the environment can modify interactions between hosts, change host and vector densities, and alter host longevity and movement [19, 20]. A study by Castillo-Neyra et al. showed that rabies transmission was spatially linked to water channels, which act as ecological corridors connecting multiple susceptible populations and facilitating pathogen spread and persistence [20]. However, with cities expanding and providing urban corridors to wildlife, pathogen persistence could become even more of an issue [20], confirming the importance of studying the effect of human perturbations on animal ecology and related implications in disease ecology.

Additionally, transmission of different zoonoses often involve multiple agents including humans and a diverse range of wild and domestic animals. In order to understand the processes behind their transmission, it is essential to clearly disentangle the role of each agent involved [19]. Due to the complexity of disease transmission and the maintenance of infection within multiple wildlife hosts, for example between bovine and badger populations in the case of bTB, the individual components of the transmission chain are often studied separately. This can limit our understanding of the subtle underlying effects explaining disease emergence and transmission. Therefore, a holistic approach is essential to develop a complete picture of the transmission dynamics of zoonotic diseases like bTB [19]; for example, recent research on rabies has shown how empirical data can be used to elucidate epidemiological dynamics [21].

However, even in cases where empirical data is used, there may be limited power, which can impact results and interpretation. In these cases, evidence from empirical data can now be boosted by mathematical simulations, which are powerful tools for predicting disease transmission trajectories [22]. Simulations of disease transmission through compartmental models (e.g., the Susceptible, Infectious, and/or Removed (SIR) model and its variations) have been used in a variety of disease systems, including the recent COVID-19 pandemic. COVID-19, however, is exceptional in the level of global concern garnered and resultant significant investment in funding. This meant that large empirical datasets were also made readily accessible, which made direct complex modelling possible [23]. Other zoonoses are typically more difficult to model this way due to the lack of empirical data on disease transmission and associated hosts [24]. Mathematical simulations, using for example SIR models, therefore create opportunities to also model these zoonoses. In addition, such simulations allow us to undertake experiments that are currently logistically unfeasible, too costly, too complex or on “unobservable” phenomena [22, 25].

As mentioned, lack of information on associated hosts and transmission pathways is often a limiting factor in modelling zoonoses and may potentially also be an issue in bTB research. Studying interactions between and within host species, as well as the role played by each host in the transmission chain, can enable us to better understand zoonotic disease dynamics. While simulations can achieve much, it is important to note that interactions amongst wild animals are heterogeneous by nature and vary significantly between different populations as well as individuals. Therefore, it is important to account for this variability to understand the mechanisms behind transmission and subsequently be able to predict and control disease spread [8]. This can be achieved by using network modelling, where heterogeneous contacts between animals can be used to simulate disease transmission [8, 24], for example using social network analysis (SNA) [8]. SNA can be beneficial for disease management since it enables us to identify “super-spreaders” (i.e., highly connected individuals) which can then be targeted for vaccination, allowing for a dramatic reduction in transmission [1, 8]. In addition, new research is looking at integrating SNA with molecular epidemiology (phylodynamics) to better estimate transmission pathways and direction of transmission between individuals [26].

Finally, it is of key importance that models of disease risk and distribution consider variances across space and time [27, 28], which enables us to identify disease clusters [5] and model host abundance [29]. As ecological processes occur at different scales (from single study sites to macroecological scales), the spatial scale used for disease distribution modelling is crucial in understanding how these processes exacerbate the spread of zoonotic diseases, such as bTB [30, 31]. Large spatial scales (i.e., global, continental) can examine the broader picture and disentangle how host abundance and abiotic factors influence disease prevalence [19]. Smaller spatial scales (i.e., country, region) can be used to examine population dynamics and pathogen genetic diversity at the local level [19]. Temporal patterns are important to consider as many zoonotic diseases show seasonal variations (e.g., Zoonotic enteric diseases such as *Salmonella* spp, *Escherichia coli*, *Giardia* spp) as well as daily variations (i.e., due to the circadian rhythm of microbes and pathogens as well as chronobiology of wildlife hosts) in their infection patterns [32–34]. It is of key importance that any gaps in bTB research pertaining to factors discussed above be identified, in order to inform future research direction.

Here, we aimed to uncover empirical and methodological gaps in the peer-reviewed literature on bTB. Our intention is to use bTB as an example of a complex multihost zoonotic disease for which recent developments with sampling design, animal monitoring tools and technology, and mathematical modelling has helped to fill the gaps in knowledge and improve our understanding and ability to combat zoonotic diseases more generally.

**Figure 1:**
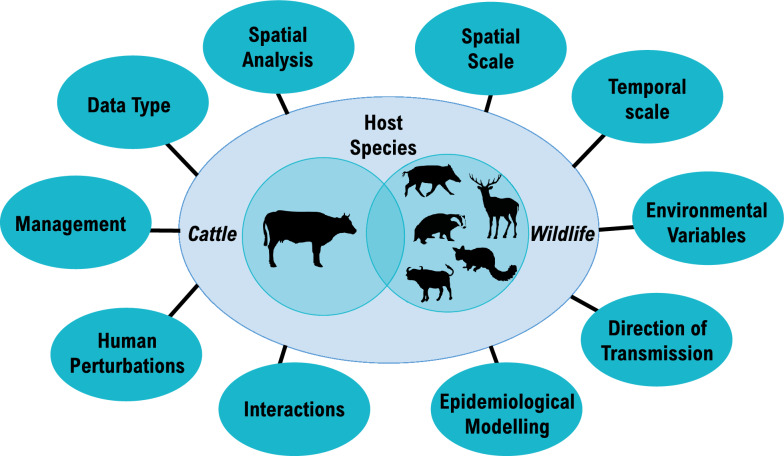
**Key host species and topics of interest we screened for in the bovine tuberculosis scientific literature published between 1981 and 2022**. bTB host species include cattle as well as a range of wild species: badger, wild boar, cervid species (with the following species identified in the literature screened: white-tailed deer, red deer, fallow deer, roe deer, wapiti elk, sika deer and muntjac deer), brush-tailed possum and wild buffalo. The circles on the outside illustrate the key information sought in peer-reviewed papers dealing with bTB, which has been expanded and clarified in Table 1: type of data collected by researchers; whether spatial analyses were carried out (i.e., in cattle and or wildlife); what type of spatial and temporal scales were considered; whether environmental variables were taken into account (i.e., environment in the farm, environment around the farm and/or weather variables); whether the methodological approach captured the direction of disease transmission; whether the study used common epidemiological modelling techniques (i.e., compartmental models, transmission rates), or whether the study included intra/interspecies interactions in their methodology (i.e., what type of interactions did they look at - e.g., direct and/or indirect, what type of equipment was used to get interactions data and what methodology was used to analyse the data); finally, if human perturbations (i.e., forest felling, culling, vaccination) were taken into account when looking at variables affecting bTB spread, and management solutions to offset the spread of bTB, if any. Animal silhouettes were downloaded from PhyloPic [134]. Cattle, cervid, brushed-tailed possum and wild boar silhouettes are under: CC0 1.0 Universal (CC0 1.0) Public Domain Dedication. Buffalo silhouette is by Jan A. Venter, Herbert H. T. Prins, David A. Balfour & Rob Slotow (vectorized by T. Michael Keesey) under: Attribution 3.0 Unported (CC BY 3.0) [135]. Badger silhouette is by Anthony Caravaggi under: Attribution-NonCommercial-ShareAlike 3.0 Unported (CC BY-NC-SA 3.0) [135]

**Figure 2:**
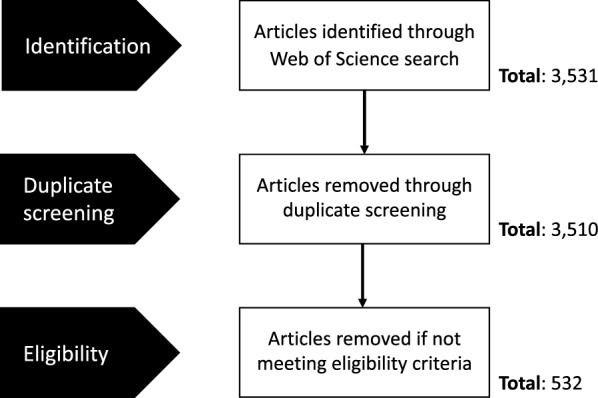
**Cascade diagram of the process used in the selection of relevant papers.**

To achieve our goal, we developed a scoping review of bTB multihost epidemiology focusing on 18 research questions (reported in Table 1 and conceptually summarised in Figure 1) regarding the type of study, whether, which and how wildlife species have been monitored, what kind of sampling designs and methodological approaches have been used, and whether epidemiological empirical data have been collected. We then gathered data from the peer-reviewed literature on the mechanisms driving inter- and intraspecies bTB transmission, looking in particular at novel and multi-disciplinary approaches. Our goal is that our work will spark renewed discussion on how to monitor and deal with zoonotic diseases, direct future research, and stimulate focused funding efforts (Figure 2).

**Table 1:** **Description of the 18 questions (and related variables) used to screen bTB papers published before July 2022**

Variables		
Variable types	Variable names	18 questions
*General characteristics*	Study type	Is the study on cattle and/or wildlife?
	Wildlife species	Which wildlife species are included in the study: i.e., cervid, badger, wild boar, brush-tailed possum, wild buffalo?
	Management	Does the study look at possible management solutions for controlling the spread of bTB from wildlife to livestock and their efficacy?
	Data type	Is the study based on empirical data and/or simulations?
*Data analysis*	Spatial analysis—cattle	Does the study conduct spatial analysis in cattle (e.g., looking at risk and probability of infection)?
	Spatial analysis—wildlife	Does the study conduct spatial analysis in wildlife (e.g., using GPS collars)? (Which does not imply testing for disease but rather whether the spatial behaviour of the potential wildlife host has been studied)
	Spatial scale	Does the study include a spatial scale and if so which type: local, regional, national and international?
	Temporal scale	What type of temporal scale does the study include, if any? Temporal scale type: daily variability, snapshot (1 point in time), intra-annual variability, interannual variability, future scenario predictions
	Farm environment	Does the data analysis include variables that explain the environment characteristics inside the farm (e.g., herd data, habitat within the farm)?
	Outside farm environment	Does the data analysis include variables that explain the environmental characteristics around the farm (e.g., habitat characteristics)?
	Weather	Does the data analysis include climate variables (e.g., rainfall, soil humidity)?
	Human perturbation	Does the data analysis include human perturbation variables (e.g., culling, road construction, forest clearfell)?
*Epidemiology*	Intra and interspecies interactions	Does the study look at interactions between animals and, if so, does it look at intraspecies or interspecies interactions?
	Interaction type	If the study looks at interactions, what type of interactions does it look at? Type of interactions: indirect or direct
	Interaction equipment	If the study looks at interactions, how was the data collected? Collection type: direct observations, technological tools (GPS, proximity loggers, camera traps), simulation
	Interaction methodology	If the study looks at interactions, what methodology was used for the data analysis? Methodology type: differential equations, social network analysis, linear models or other (e.g., t-test/ANOVA, stochastic models)
	Direction	Does the study include a direction of transmission (e.g., using whole genome sequencing of genetic samples) and/or a clear and direct path of contacts between animals (e.g., using a social network)?
	Epidemiological modelling	Does the study look at compartmental models (i.e., SIR) and/or transmission rates?

The variables have been divided into three groups depending on the question type. Variables looking at general aspects of the paper (e.g., type of host species included in the study) were grouped as *general characteristics*. Variables concerning the type of data analysis conducted in the studies were grouped as *data analysis*. Finally, variables focused on the type of epidemiological analysis conducted in the papers of interest were grouped as *epidemiology*.

## Methods

We conducted a scoping review (as per PRISMA guidelines) [35] by sourcing peer-reviewed papers using Web of Science (Clarivate, 2021 Online Version) focusing on bovine tuberculosis, and more specifically its most common cause, *Mycobacterium bovis*, in cattle and several key wildlife hosts. The search terms and list of articles have been summarised in Additional file 1. We identified 3531 potentially relevant papers (i.e., the search included all years of publication) which were uploaded and screened for duplicates using EndNote (Clarivate, Version 20.1.0.15341)(Figure 2). Relevant articles were then selected using a PEO (Population, Exposure, Outcomes) eligibility criterium structure [36]. The aim of the PEO is to identify articles of interest by selecting the “Population” (i.e., the subject being affected by the disease/health condition) for a particular “Exposure” (i.e., a disease/health condition) and either a particular “Outcome” or “Themes’’ to examine [36, 37]. The PEO eligibility criterium was chosen since it was in line with the recommendations given for scoping reviews that target literature on etiology and risk factors, such as a particular disease. We decided to use a modified version of the PEO framework structure which also includes themes of interest as potential “Outcomes” [37], as summarised in Table 2 to aid reproducibility. All papers that did not meet the eligibility criteria listed in Table 2 were removed (Figure 2). The papers were screened by one researcher who coded 18 variables (stored in an excel spreadsheet) to answer the questions of interest summarised in Table 1. The results were then imported and plotted using *ggplot2* in R version 4.1.1 [38].

**Table 2:** **Eligibility criteria used in the selection of papers for the systematic scoping review**

Eligibility criteria			
PEO criteria	Explanation	Scoping review criteria	Eligible paper
*Population*	Who is the focus of the scoping review?	Cattle, badger, deer, elk, wild boar, wild buffalo, brushed-tailed possum (i.e., known bTB hosts)	Contains at least one of the species of interest as a study species
*Exposure*	What issue is the focal point of the scoping review?	Bovine tuberculosis (i.e., *Mycobacterium bovis*)	Focal point of the paper must be bovine tuberculosis, more specifically the most common cause: *Mycobacterium bovis*
*Outcomes/Themes*	What themes, in relation to the issue, will the scoping review focus on?	• *Risk*: Does the study assess or evaluate potential risks of spreading bTB and its drivers (biotic and/or abiotic factors)? • *Management*: Does the study look at potential management solutions for controlling the spread of bTB from wildlife to livestock and their efficacy? • *bTB occurrence*: Does the study look at the presence of bTB in cattle and/or reservoir hosts at a spatial and/or temporal level? • *bTB spread*: Does the study look at the mechanisms behind the active spread of bTB between individuals of the same species and/or interspecies transmission?	Focuses on at least one of the themes of interest

The criteria follow a PEO (Population, Exposure, Outcomes/Themes) structure.

## Results

**Figure 3:**
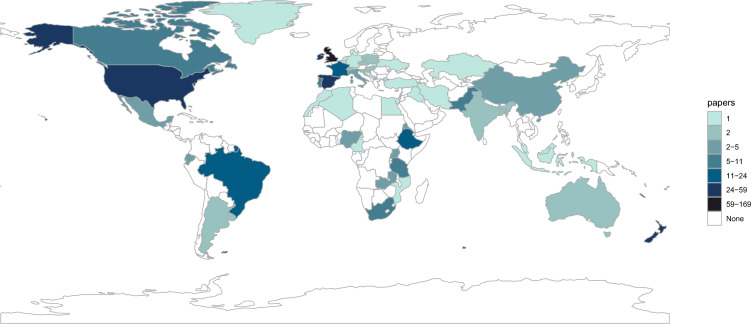
**World map showing number of papers screened per country**. Number of papers per continent: Europe (303), Africa (68), Oceania (60), North America (53), South America (29), Asia (26).

Our results are based on 532 peer-reviewed papers published between 1981 and 2022. The study location of the papers was representative of 6 continents and 52 different countries (Figure 3). The continent with the highest number of studies on bTB is Europe (*n* = 303, 169 of which were from the UK), significantly higher than those carried out in much larger continents such as Africa, Asia, and both Americas (Figure 3). We screened all papers for 18 different variables (addressing our 18 questions, see Table 1) which we summarised in the following section under the heading: 3.1 general characteristics (Sub-headings: “Study species and wildlife species”; “Management and data type”), 3.2 data analysis (Sub-headings: “Spatial analysis, spatial scale and temporal scale”; “Farm environment and human perturbations”), and 3.3 epidemiological analysis (Sub-headings: “Intra- and interspecies interactions”, “Direction of transmission and compartmental models”). Note that most plots presented below have a sample size of *n* = 532, corresponding to the number of papers screened, with a few exceptions where this sample size is higher (for example, in relation to temporal scale included in the study, if a paper reported multiple temporal scales, therefore contributing to multiple levels of a category) or lower (for example, in relation to epidemiology, where variables of interest were analysed only in the subset of papers describing studies that included epidemiological interactions).

### General characteristics

#### Study species and wildlife species

**Figure 4:**
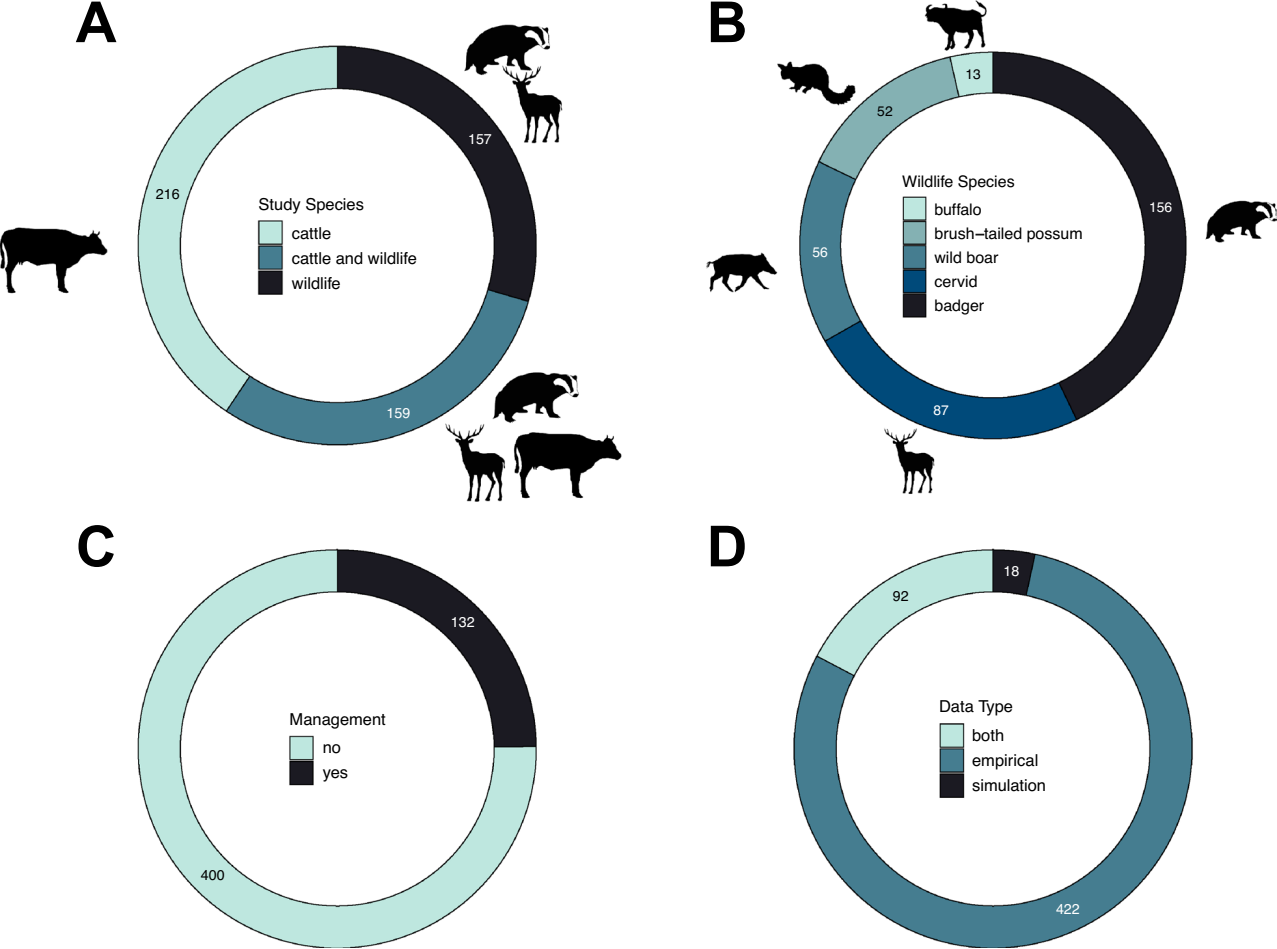
**Species, data and study type**. Number of papers screened and reporting data on **A** study species type (whether the study was on cattle and/or wildlife), **B** wildlife species, **C** management (whether a paper investigated potential management solutions and their efficacy), **D** and data type. Animal silhouettes were downloaded from PhyloPic [134]. Cattle, cervid, brushed-tailed possum and wild boar silhouettes are under: CC0 1.0 Universal (CC0 1.0) Public Domain Dedication. Buffalo silhouette is by Jan A. Venter, Herbert H. T. Prins, David A. Balfour & Rob Slotow (vectorized by T. Michael Keesey) under: Attribution 3.0 Unported (CC BY 3.0) [135]. Badger silhouette is by Anthony Caravaggi under: Attribution-NonCommercial-ShareAlike 3.0 Unported (CC BY-NC-SA 3.0) [135].

We found that 41% of bTB papers focused on cattle only, whereas 30% of them included both cattle and wildlife species and 29% targeted only wildlife species (Figure 4A). Among those papers reporting wildlife data, we found that the European badger attracted most research effort (50% of wildlife studies), followed by cervid species (28%: 13% red deer, 11% white-tailed deer, 5% fallow deer, 3% roe deer, 2% wapiti elk, from hereinafter referred to as simply elk, and < 1% of studies including sika and muntjac deer), wild boar (18%), brushed tailed possum (17%) and buffalo (4%) (Figure 4B).

#### Management and data type

Our results highlighted that only 25% of the studies dealt with management solutions (Figure 4C). Management strategies mainly included culling (18%) or vaccination (6%), with 5% looking at other strategies (e.g., fencing, sterilisation). We also found that most papers gathered original empirical data (79%), and papers only using simulations were limited (4%), with a remaining 17% of papers combining empirical data and simulations (Figure 4D).

### Data analysis

#### Spatial analysis, spatial scale and temporal scale

**Figure 5:**
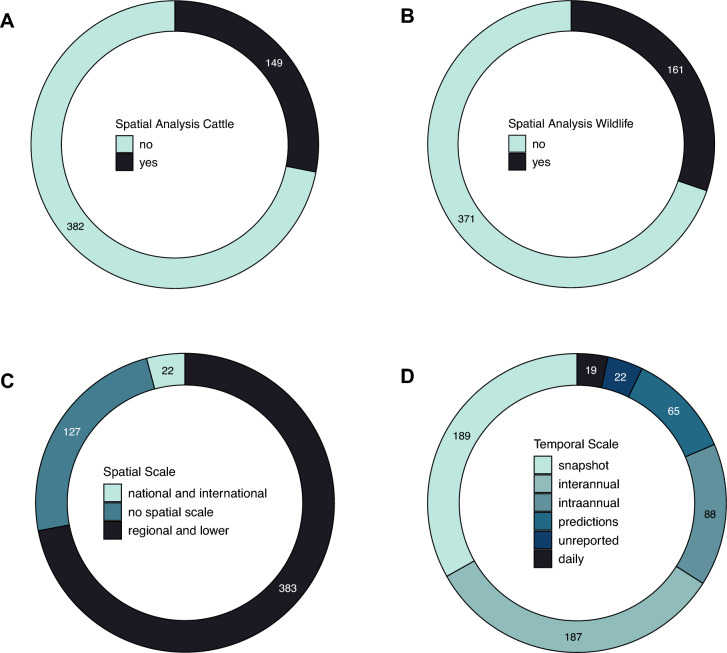
**Spatial and temporal analysis**. Number of papers screened and reporting data on **A** spatial analysis of cattle (whether the study included any type of spatial analysis), **B** spatial analysis of wildlife, **C** spatial scale, and **D** temporal scale.

We found that the majority of papers did not include any spatial analysis. Those that did focused on spatial patterns in wildlife (30%, Figure 5B) slightly more than cattle (28%, Figure 5A). Among the 149 papers that investigated spatial analysis in cattle, 58% looked at bTB risk and probability of infection; 16% looked at cattle interactions with wildlife, 13% analysed the spatial distribution of bTB positive biological samples, 11% investigated cattle movement outside the farm. Interactions between farm animals and cattle movement inside the farm were included in 5% and 1% of papers, respectively. Among the 161 studies which investigated spatial behaviour in wildlife (Figure 5B), analysis was undertaken using a variety of methodologies; direct observations (36%), satellite GPS telemetry (19%), spatial patterns predicted by future scenarios modelling or mathematical simulations (19%), genetic samples (11%), camera traps (7%), proximity loggers (4%) and indirect observations (e.g., faecal samples for population density estimations; 1%).

We also found that most papers included spatial scales at the regional level or smaller (72%), with less than 4% papers looking at national and/or international spatial scales (Figure 5C). In regard to temporal scales, 36% of the studies considered interannual variability, whereas 17% tackled intra-annual variability. Thirty-six percent of the studies did not analyse any intra- or interannual temporal variability (Figure 5D). Only 4% of the studies looked at fine-scale variability (e.g., days), whereas in a few instances the year of study was not reported at all (4%). Finally, 12% of papers included predictions for temporal patterns into future scenarios.

#### Farm environment and human perturbations

**Figure 6:**
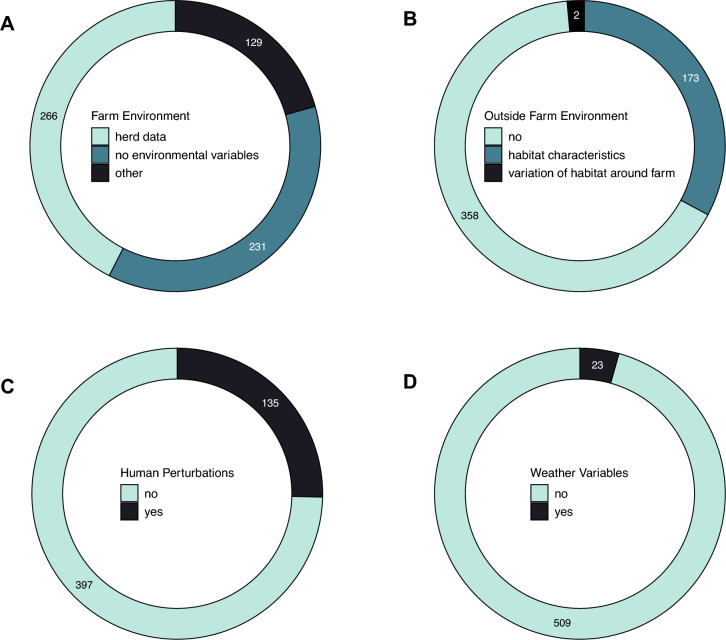
**Environmental variables**. Number of papers screened and reporting data on **A** in-farm environment (whether the paper analysis included variables explaining environmental characteristics inside the farm), **B** outside farm environment, **C** human perturbations (whether the paper analysed the effect of human disturbances on bTB transmission dynamics), and **D** weather variables.

When looking at farm characteristics, 50% of the studies included some type of herd data (e.g., herd size, bTB history), with 44% not including any type of in-farm environmental variables (Figure 6A) and 24% of papers incorporating other types of farm characteristics. These included environmental conditions on the farm (e.g., natural habitats, land-fragmentation; 13%), farm location in respect to other farms (11%) and farm location in respect to wildlife (6%).

Environmental conditions outside the farm were included in 33% of the papers’ data analysis (Figure 6B). These studies mainly looked at habitat characteristics around the farm (e.g., wildlife presence, natural habitats), with two papers also including variables focusing on habitat variation (e.g., forest clearfell, new artificial plantations). We also looked at weather variables (e.g., temperature, rainfall) and observed that 4% of papers included these as part of their analysis (Figure 6D). Finally, 25% of the papers screened included human perturbation variables with the vast majority looking at the effect of vaccination and culling on transmission dynamics (Figure 6C).

### Epidemiological analysis

#### Intra- and interspecies interactions

**Figure 7:**
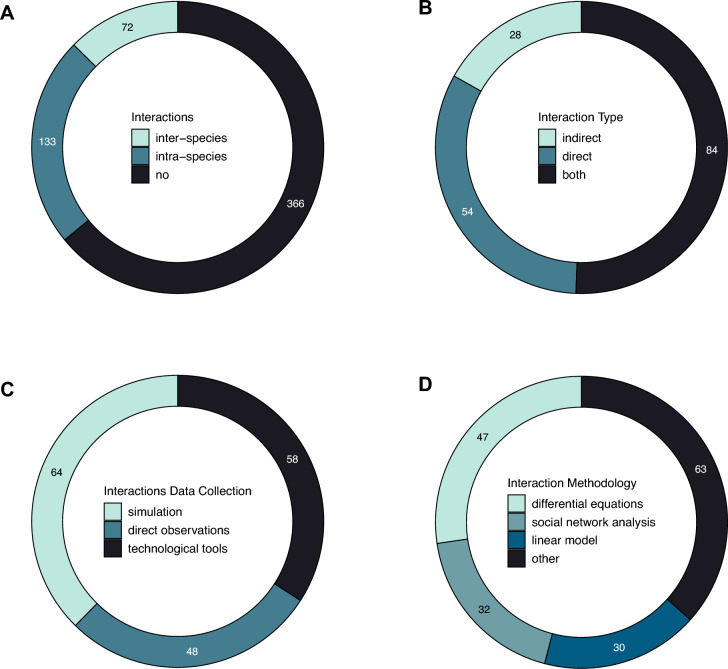
**Interaction analysis**. Number of papers screened and reporting data on **A** interactions (inclusion of interaction analysis i.e., intra- and/or interspecies interactions), **B** interaction type, **C** the way interactions were monitored, and **D** interaction data analysis statistical approach.

We found that most papers (69%) did not include an analysis on interactions, with 25% of papers looking at intraspecies transmission and 14% at interspecies transmission (Figure 7A). Among the interaction studies, 33% included direct interactions, 17% included indirect interactions and 51% included both (Figure 7B). In addition, interaction data were mostly collected using simulations (39%), followed by technological tools (29%; e.g., GPS, proximity loggers, camera traps), and direct observations (23%), with genetic sampling used in 7% of papers (Figure 7C). The methodology used to analyse interaction data also varied between papers with 28% of papers using differential equations (e.g., SIR models, discreate models), 19% social network analysis, 18% linear models (i.e., including generalized mixed models as well as simple linear regressions) and 38% using a variety of statistical techniques (e.g., t-test/ANOVA, stochastic models) (Figure 7D).

#### Direction of transmission and compartmental models

**Figure 8:**
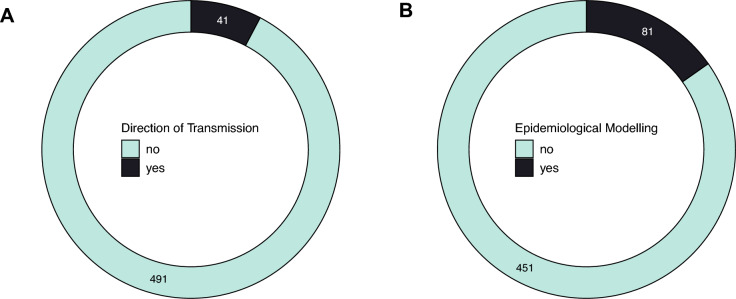
**Epidemiological analysis**. Number of papers screened and reporting data on **A** direction of transmission (whether this was analysed in the paper, e.g., transmission across species), **B** epidemiological modelling (i.e., papers included compartmental models and/or transmission rates in the analysis).

We found that a limited proportion of the papers (8%) included direction of transmission in their analysis (Figure 8A). We also found that epidemiological modelling techniques (e.g., compartmental models and transmission rates) were adopted in 15% of the studies (Figure 8B).

## Discussion

In this review we found that there has been significant research focusing on the badger-cattle bTB episystem. We acknowledge, however, that we also found a very limited number of studies on other episystems [39–41]. Our spatially-explicit overview of bTB research efforts (Figure 3) highlights how the badger-cattle episystem has been the focus of most research done to date, highlighting a huge amount of money and research effort on bTB transmission dynamics across Europe and particularly in Britain and Ireland. However, there has been far less attention given on other multi-host episystems of countries in southern Africa, Asia and both South and North America. We believe we have more to learn from these chronically understudied systems.

Our scoping review found a limited number of studies focusing on management solutions and their efficacy, with very few looking at modelling exit strategies [42, 43]. This is due to the paucity of studies using mathematical simulations, not only to better understand and predict possible outputs of management solutions, but also to explore long-term bTB dynamics under different scenarios (e.g. [44, 45]). Only a small number of studies have looked at the effect of human disturbances on the spread of bTB in wildlife host species, and this knowledge gap needs to be tackled as we are aware that human perturbations may exacerbate zoonotic outbreaks and spread [46–48]. Most of the studies we reviewed have focused on the effect of badger vaccination and culling on bTB dynamics with only three studies looking at how other human perturbations may affect these dynamics [49–51]. Additionally, only two focused on the effect of habitat change (e.g., clearfell forest operations) on bTB breakdowns [52, 53]. Finally, we observed that there is only a few studies looking at the effect of weather variables (i.e., rainfall, soil humidity, temperature etc.) on bTB spread or risk [54]. This is especially important when considering wildlife-cattle transmission since it is now thought to occur also through environmental sources [55].

We have carefully evaluated the outcome of our scoping review, and in the following sections we have summarised data types and methodological approaches which, we believe, could contribute to gaining further insights into bTB epidemiology. Based on our review, we have identified a significant gap when it comes to prediction and simulation models, which would be a useful tool for managers to assess disease risk under different land use and climate change scenarios. Another major gap is the lack of integration between empirically-informed tactical (short-term decision support) and strategic (larger spatial scales and longer term) models being used concurrently in single studies (though we do note that there are exceptions, for instance Brooks-Pollock et al. [56]). Future research should include compartmental models fitted across space, linked via meta-populations and/or real-landscape multi-host episystems; or agent-based models (ABMs) with empirical data feedback loops. We describe such modelling approaches and their prerequisites in the following sections, beginning with data and monitoring programs, and we continue with recent advances in technology, mathematical tools and analytical solutions.

### Empirical data and long-term monitoring programs: involving stakeholders and setting up fixed long-term monitoring stations across large spatial scales

As good quality data is required to generate informed strategies on wildlife interventions, we need reliable data sources to model spatial distribution and abundance of the host species involved in transmission. In reference to the badger-cattle bTB episystem in western Europe, both badgers (with several examples among the literature: [57, 58]), and cattle [59] have been extensively monitored. However, in some populations, it is possible that deer and wild boar may also play a role in the local spread and maintenance of infection [60]. In Britain and Ireland, the significance of deer as a wildlife host impacting bTB epidemiology has been uncertain [61]. However, recent research is starting to uncover the role deer may play at local scales where conditions favour the transmission between badgers-deer-cattle [62]. There could be opportunities to gather data in collaboration with hunters (as has occurred in France [63] and Spain [64], for example) to have access to a high number of deer samples, within and across countries (large spatial scales) and across years (long-term temporal scales). Involving stakeholders like hunters may provide the unique opportunity to collect pictures of clearly infected animals (e.g., small to large white, tan, or yellow lesions on the lungs, rib cage, or in the chest cavity) - to be submitted via smartphone applications (see [65]). These stakeholders may also gather biological samples to be collected by government officials at ad hoc collecting centres. This type of information would boost opportunities for monitoring the dynamics of the disease across multiple spatio-temporal scales and in relation to bTB occurrence in the two other hosts in the system (badgers and cattle). The ability to involve stakeholders across large spatial scales (e.g., hunters, farmers, foresters) may help to establish systematic, relatively inexpensive, and long-term monitoring programmes. These can provide species presence-only and presence-absence data for Bayesian species and disease distribution models (described in Sect. "Modelling and mathematical simulations: social network analysis Bayesian species distribution models, and agent based models"), allowing managers to access up to date risk scenarios. This approach can also highlight hotspots of disease outbreak that could drive focused longitudinal studies using satellite telemetry on multiple species simultaneously. This would enable us to better disentangle species overlaps and contact rates [66, 67]. The role of stakeholders/citizen scientists in this bTB example could be confirming infection, which is almost never inexpensive, although there is the hope that cheaper field tests will be released in the next decade. The veracity of the data collected and level of engagement from stakeholders/citizen could also be a problem which needs to be taken into consideration. For the time being, a well distributed number of samples could be collected from hunters to cover large areas systematically and limit the costs required for testing.

When it comes to establishing long-term monitoring programs, fixed long-term sampling stations across large-spatial scales can capture wildlife population spatio-temporal dynamics. This can, on one hand, provide data on occurrence, relative density, and spatio-temporal overlaps of the host species and, on the other hand, gather key empirical data required to parameterise mathematical simulations. Camera traps are a popular and effective tool for estimating state variables of wildlife populations [68]. For ungulates, they have successfully been used to understand temporal behaviour (e.g., diel activity patterns, [69]), spatial behaviour (e.g., occupancy, [70]), and abundance (e.g., density, [71]). Camera traps have been used for quantifying temporal and spatial overlap of wild ungulates with domestic animals in open systems [72, 73] with varying results [74]. Kukielka et al. demonstrated their use in identifying hotspots of indirect wildlife–livestock overlap for the prevention of bTB crossover [72]. For wildlife, especially ungulates, camera traps offer powerful monitoring solutions not only to measure abundance and spatial overlap, but also to understand behavioural dynamics that may align closely with disease risk. An example is the use of camera traps to individually recognise animals, which has been shown to be possible in a recent study by Hinojo et al. [75]. They demonstrated how roe deer (*Capreolus capreolus*) antler shapes could be used to identify distinct individuals. This data could be used to obtain better estimates of abundance as well as to build wildlife social networks (which will be discussed in more detail in Sect. "Modelling and mathematical simulations: social network analysis, Bayesian species distribution models, and agent based models") and therefore provide information on contact rates between and within species. The parameters from these analyses would be useful as an input for mathematical simulations to help better understand disease transmission dynamics in wildlife populations.

The use of camera traps as well as satellite telemetry can be quite challenging to use in developing countries since they can be extremely expensive (satellite telemetry, in particular) as well as difficult to use when collecting data in remote locations (camera traps, in particular). In addition, the invasive nature of satellite telemetry - which requires trapping animals - often makes it hard to collect data from enough individuals from an ethical, logistical and administrative points of view. Therefore, to improve our understanding of episystems in developing nations, advances in non-invasive diagnostic techniques and eDNA (i.e., a genetic sampling technique that uses environmental sources - such as water and soil - to extract genetic information used for biosecurity and biomonitoring purposes) are essential [76–79]. An example of a widely used non-invasive sampling technique is faecal sampling [76–81]. Faecal samples are a relatively inexpensive way of monitoring diseases and health status in wildlife species. It is also possible to collect a high number of samples in a short period of time, which is especially important for long-term monitoring programs of wildlife hosts. Collecting eDNA can be even faster and is especially useful for long term spatio-temporal dynamics of infectious pathogens at the wider scale, which can improve the monitoring of zoonoses at the country and continental level [77].

However, timing is key when monitoring diseases as infectious pathogens can mutate and be rapidly transmitted between wildlife, humans, and domestic animals, with potentially devastating impacts on human health and animal welfare. Therefore, novel and rapid genetic techniques, such as culture-free pathogen genetic sequencing [82], can greatly benefit disease surveillance by decreasing the time needed to sequence pathogens and, consequently, the time needed to make essential ecological management decisions and activate public health responses. In addition, these new sequencing technologies can be very useful during wildlife field studies in isolated areas since they can be rapidly deployed and need limited laboratory equipment for processing [82]. In addition, when monitoring zoonosis such as bTB and collecting related data (invasively or not) it is important to recall the characteristics of the bacterium itself, *Mycobacterium bovis*. For example, different lineages exist across the globe [83] with different strains potentially showing different evolutionary [clock] rates. This greatly affects the rate at which the bacterium needs to be monitored among countries, and we believe that faster sequencing technologies will be of great help in tracking the evolution and spread of different lineages, informing adaptive management of bTB (and zoonosis in general) at the local level.

### Recent advances with technology can help to gather data for mathematical simulations: interindividual variability within animal populations and human socio-economic factors matter and should be taken into account

Animal-attached sensors, i.e., biologging [84, 85], can allow us to disentangle animal behaviour and the movement patterns that promote disease transmission. GPS units are the most widely used of these sensors, providing data on animal space use. Proximity sensors can detect when two or more sensor-equipped animals interact and can be used to detect direct encounters which may result in disease transmission. Collars with both GPS units and proximity sensors have been used concurrently on badgers and cattle uncovering that, while badgers show a habitat preference for cattle pastures, there were rare to no direct contacts between the two species [86, 87]. This indicates that environmental transmission may play an important role in the case of bTB [87]. As such, proximity sensors allow insights which are not obtainable through investigating shared space use alone. When the disease state of an individual is known, proximity sensors can also provide information on if and how the duration of exposure to said individual affects transmission rate to other members of the population [88]. Other biologging sensors, including accelerometers, magnetometers, and gyroscopes, are used to classify distinct behaviours from logged datasets [85]. Behaviour classification allows activity budgets to be built so that behaviours which increase the likelihood of acquiring or transmitting pathogens can be detected and mapped in the landscape. Accelerometers have also been used to compare micro-movements in diseased and healthy animals, with diseased animals exhibiting differences in posture, gait dynamism (e.g., the “bounce” in subsequent walking steps) and energy levels [89]. Monitoring such micro-movements in cattle could act as a warning sign to test herds for bTB when signs of illness are detected, e.g. by adapting existing systems in place to monitor lameness through accelerometry [90]. These effects of disease on the internal state of animals yield important insights into how disease status impacts animal movement patterns and therefore disease spread.

Biologging and satellite telemetry monitoring can, on one hand, provide answers aimed at understanding the transmission dynamics within multi-host disease systems [87, 91, 92] and, on the other hand, provide highly valuable empirical data that are strongly needed by parameter hungry mathematical simulations [88]. However, when tracking animals, special care should be taken to understand the behaviour of those animals that we are monitoring, and specifically whether we are following a bolder subset of the overall population that are easier to trap. This applies also to where we study animals which will provide empirical data for mathematical simulations, because behaviour and movement ecology may vary significantly depending on the level of human disturbance. We are aware that tracking multiple individuals of multiple species can be expensive and not accessible unless large amounts of funding is available. However, recent technological advances with satellite telemetry using LoRaWAN transmission technology [93, 94] have been developed to monitor livestock at affordable prices (e.g. less than 100 euros for 1 GPS unit), opening up new opportunities for extensive monitoring programmes in wildlife, within and across species.

The concept of One Health has highlighted the role that human activities play in the spread of zoonotic diseases [95]. For example, urbanisation, improper waste disposal, and the intentional feeding of wildlife have been shown to result in wildlife movement into human-dominated areas [7], which may facilitate disease transfer to humans and other animal communities [96]. However, evidence has shown that only a select proportion of individuals within wildlife populations will engage in interactions with humans [97] or utilise these human-dominated areas [7, 98]. Individual variation in movement patterns [99], sociability [100], and immunological defence [101], among others, impacts disease transmission and spread [102]. There is also evidence that certain behavioural types have higher infection rates than others (e.g. [103, 104]), although the causal direction may be difficult to determine since infections also alter host behaviour [103, 105]. Regardless, to gain a more complete understanding of disease spread, future studies should incorporate this individual variation. These studies often utilise direct behavioural observations, since these are an invaluable data source that can be used to determine which individuals in a known population are more likely to engage in close-contact interactions with humans [97] or access human areas (e.g., farmland) [106]. This can provide us with information on which individuals in a population may be at “higher risk” of transferring disease to humans or to other animal populations.

Nevertheless, considering human behaviour is also fundamental in infectious disease transmission. The One Health definition has changed in 2022 accordingly and now it includes the importance of society and its diversity in values and beliefs in effectively fighting zoonoses [107, 108]. Collaboration between scientific disciplines is not enough to improve current and emerging infectious disease transmission. It is fundamental that community members and expertise at every level, from village to continent, be included if we wish to equitably improve human health and animal welfare [107]. In this way we may also improve the effectiveness of disease management solutions by tailoring them to communities instead of trying to use the same solutions in different areas without taking into account socio-economical differences.

### Modelling and mathematical simulations: social network analysis, Bayesian species distribution models, and agent based models

Social network analysis (SNA) is a powerful tool in uncovering the causes and consequences of disease transmission within animal communities [109, 110]. In the past decade SNA has mainly focused on understanding contact and transport networks of cattle and livestock movements, as well as wildlife movements [111–113]. Nonetheless, it could be expanded to better unravel the dynamics of disease transmission between wildlife populations and livestock [110]. Unlike in domestic cattle, the movements and interactions of wildlife can be challenging to track. As a result, a small proportion of individuals are typically monitored using biologging and satellite telemetry, as discussed earlier. Recent advances in statistical analysis of social networks have paved the way to obtain better inferences from limited data [114–116]. The first step is to identify the network metrics affecting disease transmission dynamics that best suits the disease system under study (e.g., transitivity, betweenness centrality) [114, 115]. Using global metrics of a social network, for example, can help estimate potential changes in the overall structure of the wildlife population. A commonly used global metric when studying disease transmission dynamics is transitivity, which represents the tendency of a population to cluster together and is considered to be negatively correlated with disease transmission rates [113]. Local network metrics, on the other hand, can help in understanding social characteristics at the individual level. A type of local metric is betweenness centrality, which represents the tendency of an individual to serve as a bridge between one part of the community and another (i.e., a community in SNA is a group of nodes, for example individual animals, with denser connections between each other compared to other nodes in the network), helping the selection of individuals to be vaccinated/removed from the population.

Once we have selected the metrics to use, they can be tested via pre-network permutations of available observations to ensure that the available data sufficiently captures non-random interactions among the animals. However, when using small samples for SNA we also must be careful on what we infer from it. Recent research [115] has shown that estimates may be inaccurate, or “noisy”, at low sample sizes. Therefore, stable metrics with respect to low sample sizes should be identified before making inferences. Research on data collected for wild ungulates [115], for example, shows that the betweenness centrality values of smaller samples remain well correlated with those in larger samples, indicating that this metric can be used even when the social network is built using a small sample of the population. Similar correlation analysis can be done for other network metrics, mainly in cases of limited data availability for disease transmission. Whenever limited animals from a population are monitored, confidence intervals around the network metrics should also be obtained to make informed decisions using statistical evidence.

Using the methodologies discussed above (see Silk et al. and Kaur et al. for more details, [113, 115]) we now have the possibility of analysing all telemetry data collected thus far on species involved in bTB transmission (e.g., badgers, wild boar; but also applicable to species from other disease systems) to test hypotheses on disease transmission dynamics. For example, we can now use these statistical techniques to better understand behavioural patterns of wildlife species, as well as comparing networks overtime and how wildlife behaviour can be affected by perturbations in the environment (e.g., climate change, land-use change or other type of anthropogenic factors) even when only limited data is available [115]. In addition, it will help in collecting future data since these methodologies can be used to estimate the minimum number of individuals needed in order to reliably build a social network, which can vary enormously depending on the scope of the project as well as the wildlife species of interest. This will, for example, help in answering specific questions regarding the role of deer species in bTB transmission by simultaneously collecting telemetry data on badgers and deer species in Ireland.

Knowing the distribution and abundance of wildlife vectors (i.e., a living agent that carries and transmits pathogens - e.g. HIV, Covid-19, bTB - to other living beings) is also essential when aiming to reduce zoonotic risk [117, 118]. To that aim, Species Distribution Models (SDMs) can be used to produce models of the distribution and abundance of species based on occurrence data [119]. In recent years spatial modelling has undergone a conceptual and technical revolution. New modelling techniques within Bayesian [120] and Machine Learning frameworks [121] allow us to develop spatially explicit models of animal abundance and distributions with unprecedented accuracy, and the improvement of computational power allows computers to rise to the challenge and cope with the high computational demands of these models. The flexibility of the new techniques allows us to use different types of data (e.g., individual tracking data, survey data, and even citizen science data) and combine them in what are called Integrated Species Distribution Models (ISDMs), while still taking into account the different observational processes of each type of data, to produce accurate models even in data scarce systems [122]. In addition, these new techniques also allow for the calculation of uncertainty in a spatially explicit manner, which will help us evaluate the quality of the models and better interpret the results. Bayesian ISDMs using INLA (i.e., Integrated Nested Laplace Approximation) [123] were used to model the distribution of red, sika and fallow deer in Ireland, which are vectors of bTB [65]. The models produced, for the first time, relative abundance and distribution maps for each species, which will be an essential tool for deer population management and thus towards bTB eradication. They are already being used to determine high sika-density areas for a pilot study on the effect of deer on biodiversity, which will provide further management tools for the overabundant deer populations in Ireland. In addition, hierarchical Bayesian models are also the basis of a new project aimed at modelling European badger sett distribution, badger density, and their body condition. These three models will be linked to bTB infection in badgers and outbreaks in cattle, in an attempt for the first time to link badger spatial ecology to bTB management and eradication in Ireland (V. Morera-Pujol 2023, personal communication).

Agent-based simulations are another useful modelling approach, or complementary tool to traditional methods, when data is limited/not available; helping elucidate transient effects of wildlife disease transmission in human-dominated landscapes [25]. These models serve as a computational laboratory that allow researchers to plug-in available real-world data and parameterise both agents (for instance, a badger) and the environment (for instance, a mosaic of natural habitats and farms). This enables researchers to empirically test if animal behaviour in response to landscape change or management interventions modulates disease risk dynamics over time and space [124]. Recent technological advancements have bolstered agent-based simulations allowing for high-resolution spatio-temporal models that incorporate geographic information systems (GIS) data to create hyper realistic environments, and machine learning algorithms to introduce cognition and applied decision making for agents. Furthermore, process-driven agent-based models (e.g., disease transmission) can be integrated into larger mechanistic agent-based models (e.g., ecosystem scale epi-dynamics) for increasingly higher-resolution models that reduce uncertainty and overly-theoretical parameterisation of model entities [25]. The development of highly-realistic agent-based simulations, parameterised with high-resolution data, for the management of bovine tuberculosis in multi-host systems can contribute to answering important policy questions and how best to select management directions. In practice, this allows for the totality of data collected in complex multi-host systems to be incorporated into a single environment where they may be measured against one another in the simulation to deduce the possible effects of each predictor. Take for example the European badger as the primary wildlife host in Ireland as a case study. Badgers are prevalent in the agroecological mosaic of natural habitats and farms in Ireland. Agent-based simulations can utilise data from badger tracking studies [51, 125, 126], habitat suitability [127], culling and vaccination programmes [128] and disturbance regimes [52, 129] to simulate badger movement and behaviour realistically. GIS data for farm location and characteristics [130], as well as ecological and environmental data streams, can then place the badger agent into a highly realistic environment to examine how these factors affect badger movement, behaviour, and other parameters, for instance, contact rates with domestic animals. Interactions between agents and the environment can be modulated by sub-models to further increase the strength of the model. For instance, weather sub-models (e.g., rainfall) may influence agricultural practice and thus contact rates, as well as the length of time *Mycobacterium bovis* persists in the environment. Alternatively, disease transmission could also be sub-modelled so that contact rates may/may not result in infection [25]. Finally, management decisions can be trialled within the simulation to see how likely decisions change the status of disease within the study system, allowing for “What if?” scenarios to play out without risk to animal or human welfare or livelihoods.

## Conclusion

Our exploration of the recent literature on multi-host bTB episystems, as an example of zoonotic One Health challenges, has revealed a significant body of work utilising a diversity of methodologies at different spatio-temporal scales and subjects (individual vs. group) levels. There was a significant bias in the literature towards one particular episystem, the badger-cattle system that predominates in north-western Europe, reflecting large financial burdens (for both governments as well as the agricultural sector) and research funding investments. Alternatively, there were comparatively less publications from the global south, especially in complex muti-host episystems in southern Africa and India. In such episystems, the cost-effective and efficient collection, collation, and use of data are essential to drive greatest added value to inform on policy options.

Given the results from our scoping review, we reflect on several areas where progress could be made. This includes the need for high-quality data on wildlife hosts, even in episystems where significant research investments have already been made. Such careful collection and utilisation of empirical data could then help feed the development of social network analyses, Bayesian distribution models and eventually mathematical and simulation-based models. Mathematical simulations, such as ABMs trained on synthetic data and parameterised by real empirical data, can answer questions that would otherwise be too costly, unethical, or both. Such models can also be used to explore different scenarios in an increasingly human-dominated world, under different levels of land-use and climate change, or with the appearance of invasive species in already complex multi-host epidemiological systems. In addition, it can help build cross-disciplinary bridges with other areas, deriving significant insights into interspecific transmission like phylodynamic modelling.

We have used our Irish experience to inspire researchers from across the globe; Ireland invests considerably in surveying, culling, and vaccinating badgers [131, 132]. However, the question remains - which applies to other countries and zoonotic episystems - should we be doing more or can we be smarter with the data we already have? We suggest the latter. Yes, there is a need to be smarter, arranging ad hoc data collections using the latest technological tools to estimate unknown or uncertain parameters. But we also have to focus our efforts on mathematical modelling (ABMs, INLA-Bayesian) to optimise our information gain from the large, high-quality datasets collected over the last few decades (and for sparser datasets, taking advantage of recently developed statistical tools for enhanced inferences, see [54, 68]). We have (almost) all the data required to parameterise simulations with significant utility: this should be one main focus in future research. We believe that, ideally, the feedback of simulation and mathematical tools to inform data collection, and the “virtuous cycle” of feeding this new data to improve the next generation of model is a priority for decision making tools for policy makers and programme managers.

### Supplementary Information

**Additional file 1.** **Search terms and list of articles**. The additional file contains the search terms used in Web of Science to identify potentially relevant articles as well as the list of all the relevant articles found after the duplicate screening process.

## Data Availability

The datasets generated and/or analysed during the current study are available in the “A scoping review on bovine tuberculosis highlights the need for novel data streams and analytical approaches to curb zoonotic diseases” repository [133].
