# Supplementary material for: A scoping review on bovine tuberculosis highlights the need for novel data streams and analytical approaches to curb zoonotic diseases
Source: Vet Res. 2024 May 21;55:64. doi: 10.1186/s13567-024-01314-w (PMC11110237; doi:10.1186/s13567-024-01314-w)
Supplement: Supplementary file 1 — Additional file 1. Search terms and list of articles. The additional file contains the search terms used in Web of Science to identify potentially relevant articles as well as the list of all the relevant articles found after the duplicate screening process. [file 13567_2024_1314_MOESM1_ESM.docx]

**Web of Science Search Terms:**

TS=((cattle OR livestock OR badger OR deer OR elk OR boar OR “feral pig” OR “wild pig” OR possum OR buffalo)AND (“bovine tuberculosis” OR “bovine TB” OR “mycobacterium bovis” OR “M. bovis”)  AND (risk* OR management OR control* OR presence OR prevalence OR occurrence OR spread* OR transmission)) Indexes=SCI-EXPANDED, SSCI, A&HCI, CPCI-S, CPCI-SSH, BKCI-S, BKCI-SSH, ESCI, CCR-EXPANDED, IC Timespan=All years

**The search above was completed on the following dates:**

- 11/3/2021
- 20/7/2022

**Below can be found the list of articles selected as relevant [1-532].**

1. Abbate JM, Arfuso F, Iaria C, Arestia G, Lanteri G (2020) Prevalence of bovine tuberculosis in slaughtered cattle in Sicily, Southern Italy. Animals, 10:11.

2. Abdelaal HFM, Spalink D, Amer A, Steinberg H, Hashish EA, Nasr EA, Talaat AM (2019) Genomic polymorphism associated with the emergence of virulent isolates of *Mycobacterium bovis* in the Nile Delta. Sci Rep 9:15

3. Abdou M, Frankena K, O'Keeffe J, Byrne AW (2016) Effect of culling and vaccination on bovine tuberculosis infection in a European badger (*Meles meles*) population by spatial simulation modelling. Prev Vet Med 125:19-30

4. Abernethy DA, Denny GO, Menzies FD, McGuckian P, Honhold N, Roberts AR (2006) The Northern Ireland programme for the control and eradication of *Mycobacterium bovis*. Vet Microbiol 112:231-237

5. Abernethy DA, Upton P, Higgins IM, McGrath G, Goodchild AV, Rolfe SJ, Broughan JM, Downs SH, Clifton-Hadley R, Menzies FD, de la Rua-Domenech R, Blissitt MJ, Duignan A, More SJ (2013) Bovine tuberculosis trends in the UK and the Republic of Ireland, 1995-2010. Vet Rec 172:14

6. Abrantes AC, Serejo J, Vieira-Pinto M (2021) The association between palmer drought severity index data and tuberculosis-like lesions occurrence in mediterranean hunted wild boars. Animals 11:8

7. Acevedo P, Prieto M, Quiros P, Merediz I, de Juan L, Infantes-Lorenzo JA, Triguero-Ocana R, Balseiro A (2019) Tuberculosis epidemiology and badger (*Meles meles*) spatial ecology in a hot-spot area in Atlantic Spain. Pathogens 8:18

8. Acevedo P, Romero B, Vicente J, Caracappa S, Galluzzo P, Marineo S, Vicari D, Torina A, Casal C, de la Fuente J, Gortazar C (2013) Tuberculosis epidemiology in islands: insularity, hosts and trade. PLoS One 8:9

9. Adkin A, Brouwer A, Simons RRL, Smith RP, Arnold ME, Broughan J, Kosmider R, Downs SH (2016) Development of risk-based trading farm scoring system to assist with the control of bovine tuberculosis in cattle in England and Wales. Prev Vet Med 123:32-38

10. Agusto FB, Lenhart S, Gumel AB, Odoi A (2011) Mathematical analysis of a model for the transmission dynamics of bovine tuberculosis. Math Methods Appl Sci 34:1873-1887

11. Alberti TS, Bruhn FRP, Zamboni R, Venancio FR, Scheid HV, Raffi MB, Schild AL, Sallis ESV (2020) Epidemiological analysis of bovine tuberculosis in the southern region of Rio Grande do Sul from 2000 to 2015. Pesq Vet Bras 40:77-81

12. Alberto JR, Aranha JM, Serejo JP, Amado A, Vieira-Pinto M (2011). The utility of GIS in studying the distribution of Bovine Tuberculosis in wild boar (*Sus scrofa*) and red deer (*Cervus elaphus*) in Central Portugal. In: Paulsenc P, Bauer A, Vodnansky M, Winkelmayer R, Smulders FJM (eds) Game meat hygiene in focus. Wageningen Academic Publishers, Wageningen

13. Aliraqi OM, Al-Jammaly MM, Alhankawe OK, Al-Farwachi MI, Dahl MO (2020) Preliminary prevalence and risk factors of *Mycobacterium bovis* in local and imported breeds of cattle and buffaloes in Mosul city, Iraq. Egyptian J Vet Sci 51:83-88

14. Allepuz A, Casal J, Napp S, Saez M, Alba A, Vilar M, Domingo M, Gonzalez MA, Duran-Ferrer M, Vicente J, Alvarez J, Muñoz M, Saez JL (2011) Analysis of the spatial variation of Bovine tuberculosis disease risk in Spain (2006-2009). Prev Vet Med 100:44-52

15. Almaw G, Conlan AJK, Ameni G, Gumi B, Alemu A, Guta S, Gebre S, Olani A, Garoma A, Shegu D, Yimesgen L, Nigussie D, Wood JLN, Abebe T, Mihret A, Berg S; ETHICOBOTS consortium (2021) The variable prevalence of bovine tuberculosis among dairy herds in Central Ethiopia provides opportunities for targeted intervention. PLoS One 16:17

16. Almaw G, Mekonnen GA, Mihret A, Aseffa A, Taye H, Conlan AJK, Gumi B, Zewude A, Aliy A, Tamiru M, Yimesgen L, Nigussie D, Wood JLN, Abebe T, Mihret A, Berg S; ETHICOBOTS consortium (2021) Population structure and transmission of *Mycobacterium bovis* in Ethiopia. Microb Genom 7:12

17. Alvarez J, Perez AM, Bezos J, Casal C, Romero B, Rodriguez-Campos S, Saez-Llorente JL, Diaz R, Carpintero J, de Juan L, Dominguez L (2012) Eradication of bovine tuberculosis at a herd-level in Madrid, Spain: study of within-herd transmission dynamics over a 12 year period. BMC Vet Res 8:8

18. Anderson DP, Gormley AM, Bosson M, Livingstone PG, Nugent G (2017) Livestock as sentinels for an infectious disease in a sympatric or adjacent-living wildlife reservoir host. Prev Vet Med 148:106-114

19. Anderson DP, Ramsey DSL, Nugent G, Bosson M, Livingstone P, Martin PAJ, Sergeant E, Gormley AM, Warburton B (2013) A novel approach to assess the probability of disease eradication from a wild-animal reservoir host. Epidemiol Infect 141:1509-1521

20. Aranha J, Abrantes AC, Goncalves R, Miranda R, Serejo J, Vieira-Pinto M (2021) GIS as an epidemiological tool to monitor the spatial-temporal distribution of tuberculosis in large game in a high-risk area in Portugal. Animals 11:14

21. Arnold ME, Courcier EA, Stringer LA, McCormick CM, Pascual-Linaza AV, Collins SF, Trimble NA, Ford T, Thompson S, Corbett D, Menzies FD (2021) A Bayesian analysis of a Test and Vaccinate or Remove study to control bovine tuberculosis in badgers (*Meles meles*). PLoS One 16:15

22. Avila LN, Goncalves VSP, Perez AM (2018) Risk of introduction of bovine tuberculosis (TB) into tb-free herds in Southern Bahia, Brazil, associated with movement of live cattle. Front Vet Sci 5:4

23. Azami HY, Ducrotoy MJ, Bouslikhane M, Hattendorf J, Thrusfield M, Conde-Alvarez R, Moriyon I, Zuniga-Ripa A, Alvaro PMM, Mick V, Bryssinckx W, Welburn SC, Zinsstag J (2018) The prevalence of brucellosis and bovine tuberculosis in ruminants in Sidi Kacem Province, Morocco. PLoS One 13:17

24. Aznar I, Frankena K, More SJ, O'Keeffe J, McGrath G, de Jong MCM (2018) Quantification of *Mycobacterium bovis* transmission in a badger vaccine field trial. Prev Vet Med 149:29-37

25. Bahiense L, de Avila LN, Bavia ME, Amaku M, Dias RA, Grisi JHH, Ferreira F, Telles EO, Goncalves VSP, Heinemann MB, Neto JSF (2016) Prevalence and risk factors for bovine tuberculosis in the State of Bahia, Brazil. Semin Cienc Agrar 37:3549-3559

26. Balseiro A, Gonzalez-Quiros P, Rodriguez O, Copano MF, Merediz I, de Juan L, Chambers MA, Delahay RJ, Marreros N, Royo LJ, Bezos J, Prieto JM, Gortázar C (2013) Spatial relationships between Eurasian badgers (*Meles meles*) and cattle infected with Mycobacterium bovis in Northern Spain. Vet J 197:739-745

27. Baptista TFS, Alves MC, Pereira-Dourado SM, Costa GM, Lopes E, Bruhn FRP, Braz MS, Rocha C (2021) Spatial and temporal analyses of culls due to bovine tuberculosis in slaughterhouses of Minas Gerais state, Brazil, 2008 to 20121. Pesq Vet Bras 41:7

28. Bar-David S, Lloyd-Smith JO, Getz WM (2006) Dynamics and management of infectious disease in colonizing populations. Ecology 87:1215-1224

29. Barasona JA, Gortazar C, de la Fuente J, Vicente J (2019) Host richness increases tuberculosis disease risk in game-managed areas. Microorganisms 7:7

30. Barasona JA, Latham MC, Acevedo P, Armenteros JA, Latham ADM, Gortazar C, Carro F, Soriguer RC, Vicente J (2014) Spatiotemporal interactions between wild boar and cattle: implications for cross-species disease transmission. BMC Vet Res 45:11

31. Barasona JA, Mulero-Pazmany M, Acevedo P, Negro JJ, Torres MJ, Gortazar C, Vicente J (2014) Unmanned aircraft systems for studying spatial abundance of ungulates: relevance to spatial epidemiology. PLoS One 9:17

32. Barasona JA, VerCauteren KC, Saklou N, Gortazar C, Vicente J (2013) Effectiveness of cattle operated bump gates and exclusion fences in preventing ungulate multi-host sanitary interaction. Prev Vet Med 111:42-50

33. Barbieri JD, de Oliveira LF, Dorneles EMS, Mota A, Goncaves VSP, Maluf PP, Neto JSF, Ferreira F, Dias RA, Telles EO, Filho JHHG, Heinemann M, Amaku M, Lage A (2016) Epidemiological status of bovine tuberculosis in the state of Minas Gerais, Brazil, 2013. Semin Cienc Agrar 37:3531-3548

34. Barlow ND (1991) Control of endemic bovine tb in new-zealand possum populations - results from a simple-model. J Appl Ecol 28:794-809

35. Barlow ND (1991) A spatially aggregated disease host model for bovine tb in New-Zealand possum populations. J Appl Ecol 28:777-793

36. Barlow ND (1993) A model for the spread of bovine tb in New-Zealand possum populations. J Appl Ecol 30:156-164

37. Barlow ND (1996) The ecology of wildlife disease control: Simple models revisited. J Appl Ecol 33:303-314

38. Barlow ND (2000) Non-linear transmission and simple models for bovine tuberculosis. J Anim Ecol 69:703-713

39. Barlow ND, Kean JM, Caldwell NP, Ryan TJ (1998) Modelling the regional dynamics and management of bovine tuberculosis in New Zealand cattle herds. Prev Vet Med 36:25-38

40. Barlow ND, Kean JM, Hickling G, Livingstone PG, Robson AB (1997) A simulation model for the spread of bovine tuberculosis within New Zealand cattle herds. Prev Vet Med 32:57-75

41. Barron ES, Swift B, Chantrey J, Christley R, Gardner R, Jewell C, McGrath I, Mitchell A, O'Cathail C, Prosser A, Ridout S, Sanchez-Cabezudo G, Smith N, Timofte D, Williams N, Bennett M (2018) A study of tuberculosis in road traffic-killed badgers on the edge of the British bovine TB epidemic area. Sci Rep 8:8

42. Barron MC, Nugent G, Cross ML (2013) Importance and mitigation of the risk of spillback transmission of *Mycobacterium bovis* infection for eradication of bovine tuberculosis from wildlife in New Zealand. Epidemiol Infect 141:1394-1406

43. Barron MC, Tompkins DM, Ramsey DSL, Bosson MAJ (2015) The role of multiple wildlife hosts in the persistence and spread of bovine tuberculosis in New Zealand. New Zealand Vet J 63:68-76

44. Barroso P, Barasona JA, Acevedo P, Palencia P, Carro F, Negro JJ, Torres MJ, Gortazar C, Soriguer RC, Vicente J (2020) Long-Term determinants of tuberculosis in the ungulate host community of Donana National Park. Pathogens 9:24

45. Basit A, Hussain M, Shahid M, Ayaz S, Rahim K, Ahmad I, Rehman AU, Hassan MF, Ali T (2018) Occurrence and risk factors associated with *Mycobacterium tuberculosis* and *Mycobacterium bovis* in milk samples from North East of Pakistan. Pakistan Vet J 38:199-203

46. Bastida AZ, Tellez MH, Montes LPB, Paniagua JNJ, Benites MEJ, Barrera GEM, Ramirez-Duran N (2017) Spatial analysis of bovine tuberculosis in the State of Mexico, Mexico. Vet Ital 53:39-46

47. Bekara ME, Courcoul A, Benet JJ, Durand B (2014) Modeling tuberculosis dynamics, detection and control in cattle herds. PLoS One 9:17

48. Bekara MEA, Azizi L, Benet JJ, Durand B (2016) Spatial-temporal variations of bovine tuberculosis incidence in France between 1965 and 2000. Transbound Emerg Dis 63:101-113

49. Belchior APC, Lopes LB, Goncalves VSP, Leite RC (2016) Prevalence and risk factors for bovine tuberculosis in Minas Gerais State, Brazil. Trop Anim Health Prod 48:373-378

50. Bentil DE, Murray JD (1993) Modeling bovine tuberculosis in badgers. J Anim Ecol 62:239-250

51. Benton CH, Delahay RJ, Robertson A, McDonald RA, Wilson AJ, Burke TA, Hodgson D (2016) Blood thicker than water: kinship, disease prevalence and group size drive divergent patterns of infection risk in a social mammal. Proc Biol Sci 283:8

52. Berentsen AR, Miller RS, Misiewicz R, Malmberg JL, Dunbar MR (2014) Characteristics of white-tailed deer visits to cattle farms: implications for disease transmission at the wildlife-livestock interface. Eur J Wildl Res 60:161-170

53. Bernini A, Bolzoni L, Casagrandi R (2019) When resolution does matter: Modelling indirect contacts in dairy farms at different levels of detail. PLoS One 14:20

54. Berrian AM, O'Keeffe J, White PW, Norris J, Litt J, More SJ, Olea-Popelka FJ (2012) Risk of bovine tuberculosis for cattle sold out from herds during 2005 in Ireland. Vet Rec 170:620-637

55. Bica RFP, Copetti MV, Brum MCS (2018) Hydatidosis, cysticercosis, and tuberculosis rates in bovine slaughtered under state sanitary inspection in Rio Grande do Sul, Brazil. Cienc Rural 48:7

56. Bicknell KB, Wilen JE, Howitt RE (1999) Public policy and private incentives for livestock disease control. AJARE 43:501-521

57. Bielby J, Donnelly CA, Pope LC, Burke T, Woodroffe R (2014) Badger responses to small-scale culling may compromise targeted control of bovine tuberculosis. PNAS USA 111:9193-9198

58. Bielby J, Vial F, Woodroffe R, Donnelly CA (2016) Localised Badger Culling Increases Risk of Herd Breakdown on Nearby, Not Focal, Land. PLoS One 11:9

59. Biffa D, Inangolet F, Bogale A, Oloya J, Djonne B, Skjerve E (2011) Risk factors associated with prevalence of tuberculosis-like lesions and associated mycobacteria in cattle slaughtered at public and export abattoirs in Ethiopia. Trop Anim Health Prod 43:529-538

60. Birch CPD, Goddard A, Tearne O (2018) A new bovine tuberculosis model for England and Wales (BoTMEW) to simulate epidemiology, surveillance and control. BMC Vet Res 14:16

61. Boadella M, Acevedo P, Vicente J, Mentaberre G, Balseiro A, Arnal M, Martinez D, Garcia-Bocanegra I, Casal C, Alvarez J, Oleaga Á, Lavín S, Muñoz M, Sáez-Llorente JL, de la Fuente J, Gortázar C (2011) Spatio-temporal trends of iberian wild boar contact with *Mycobacterium tuberculosis complex* detected by ELISA. Ecohealth 8:478-484

62. Boadella M, Vicente J, Ruiz-Fons F, de la Fuente J, Gortazar C (2012) Effects of culling Eurasian wild boar on the prevalence of *Mycobacterium bovis* and Aujeszky's disease virus. Prev Vet Med 107:214-221

63. Bohm M, Hutchings MR, White PCL (2009) Contact networks in a wildlife-livestock host community: identifying high-risk individuals in the transmission of bovine tb among badgers and cattle. PLoS One 4:12

64. Bohm M, Palphramand KL, Newton-Cross G, Hutchings MR, White PCL (2008) Dynamic interactions among badgers: implications for sociality and disease transmission. J Anim Ecol 77:735-745

65. Bohm M, Palphramand KL, Newton-Cross G, Hutchings MR, White PCL (2008) The spatial distribution of badgers, setts and latrines: the risk for intra-specific and badger-livestock disease transmission. Ecography 31:525-537

66. Borja E, Borja LF, Prasad R, Tunabuna T, Toribio J (2018) A retrospective study on bovine tuberculosis in cattle on Fiji: study findings and stakeholder responses. Front Vet Sci 5:13

67. Bouchez-Zacria M, Courcoul A, Durand B (2018) The distribution of bovine tuberculosis in cattle farms is linked to cattle trade and badger-mediated contact networks in South-Western France, 2007-2015. Front Vet Sci 5:12

68. Bouchez-Zacria M, Courcoul A, Jabert P, Richomme C, Durand B (2017) Environmental determinants of the *Mycobacterium bovis* concomitant infection in cattle and badgers in France. Eur J Wildl Res 63:12

69. Bradshaw CJA, McMahon CR, Miller PS, Lacy RC, Watts MJ, Verant ML, Pollak JP, Fordham DA, Prowse TAA, Brook BW (2012) Novel coupling of individual-based epidemiological and demographic models predicts realistic dynamics of tuberculosis in alien buffalo. J Appl Ecol 49:268-277

70. Brook RK, McLachlan SM (2009) Transdisciplinary habitat models for elk and cattle as a proxy for bovine tuberculosis transmission risk. Prev Vet Med 91:197-208

71. Brook RK, Vander Wal E, van Beest FM, McLachlan SM (2013) Evaluating use of cattle winter feeding areas by elk and white-tailed deer: Implications for managing bovine tuberculosis transmission risk from the ground up. Prev Vet Med 108:137-147

72. Brooks-Pollock E, Roberts GO, Keeling MJ (2014) A dynamic model of bovine tuberculosis spread and control in Great Britain. Nature 511:228-231

73. Brooks-Pollock E, Wood JLN (2015) Eliminating bovine tuberculosis in cattle and badgers: insight from a dynamic model. Proc Biol Sci 282:8

74. Broughan JM, Maye D, Carmody P, Brunton LA, Ashton A, Wint W, Alexander N, Naylor R, Ward K, Goodchild AV, Hinchliffe S, Eglin RD, Upton P, Nicholson R, Enticott G (2016) Farm characteristics and farmer perceptions associated with bovine tuberculosis incidents in areas of emerging endemic spread. Prev Vet Med 129:88-98

75. Brown E, Marshall AH, Mitchell HJ, Byrne AW (2019) Cattle movements in Northern Ireland form a robust network: implications for disease management. Prev Vet Med 170:9

76. Brunton LA, Alexander N, Wint W, Ashton A, Broughan JM (2017) Using geographically weighted regression to explore the spatially heterogeneous spread of bovine tuberculosis in England and Wales. SERRA 31:339-352

77. Brunton LA, Donnelly CA, O'Connor H, Prosser A, Ashfield S, Ashton A, Upton P, Mitchell A, Goodchild AV, Parry JE, Downs SH (2017) Assessing the effects of the first 2years of industry-led badger culling in England on the incidence of bovine tuberculosis in cattle in 2013-2015. Nat Ecol Evol 7:7213-7230

78. Buyuk F, Bozukluhan K, Saglam AG, Gokce G, Celebi O, Celik E, Kiziltepe S, Coskun MR, Otlu S, Sahin M (2017) The prevalence estimates of *Mycobacterium bovis* infection in cattle with ELISA. J Hellenic Vet Med Soc 68(4):541-546

79. Byrne AW, Barrett D, Breslin P, Madden JM, O'Keeffe J, Ryan E (2021) future risk of bovine tuberculosis (*Mycobacterium bovis*) breakdown in cattle herds 2013-2018: a dominance analysis approach. Microorganisms 9:12

80. Byrne AW, White PW, McGrath G, O'Keeffe J, Martin SW (2014) Risk of tuberculosis cattle herd breakdowns in Ireland: effects of badger culling effort, density and historic large-scale interventions. BMC Vet Res 45:10

81. Byrom AE, Anderson DP, Coleman M, Thomson C, Cross ML, Pech RP (2015) Assessing movements of brushtail possums (*Trichosurus vulpecula*) in relation to depopulated buffer zones for the management of wildlife tuberculosis in New Zealand. PLoS One 10:14

82. Caley P (1996) Is the spatial distribution of tuberculous possums influenced by den ''quality''? New Zealand Vet J 44:175-178

83. Caley P, Coleman JD, Hickling GJ (2001) Habitat-related prevalence of macroscopic *Mycobacterium bovis* infection in brushtail possums (*Trichosurus vulpecula*), Hohonu Range, Westland, New Zealand. New Zealand Vet J 49:82-87

84. Caley P, Hickling GJ, Cowan PE, Pfeiffer DU (1999) Effects of sustained control of brushtail possums on levels of *Mycobacterium bovis* infection in cattle and brushtail possum populations from Hohotaka, New Zealand. New Zealand Vet J 47:133-142

85. Campbell EL, Byrne AW, Menzies FD, McBride KR, McCormick CM, Scantlebury M, Reid N (2019) Interspecific visitation of cattle and badgers to fomites: A transmission risk for bovine tuberculosis? Nat Ecol Evol 9:8479-8489

86. Campbell EL, Byrne AW, Menzies FD, Milne G, McBride KR, McCormick CM, Scantlebury DM, Reid N (2020) Quantifying intraherd cattle movement metrics: Implications for disease transmission risk. Prev Vet Med 185:7

87. Campbell EL, Menzies FD, Byrne AW, Porter S, McCormick CM, McBride KR, Scantlebury DM, Reid N (2020) Grazing cattle exposure to neighbouring herds and badgers in relation to bovine tuberculosis risk. Res J Vet Sci 133:297-303

88. Cano-Manuel FJ, Lopez-Olvera J, Fandos P, Soriguer RC, Perez JM, Granados JE (2014) Long-term monitoring of 10 selected pathogens in wild boar (*Sus scrofa*) in Sierra Nevada National Park, southern Spain. Vet Microbiol 174:148-154

89. Cardenas NC, Pozo P, Lopes FPN, Grisi JHH, Alvarez J (2021) Use of network analysis and spread models to target control actions for bovine tuberculosis in a state from Brazil. Microorganisms 9:20

90. Carneiro PAM, Takatani H, Pasquatti TN, Silva C, Norby B, Wilkins MJ, Zumarraga MJ, Araujo FR, Kaneene JB (2019) Epidemiological study of *Mycobacterium bovis* infection in buffalo and cattle in Amazonas, Brazil. Front Vet Sci 6:9

91. Caron A, Miguel E, Gomo C, Makaya P, Pfukenyi DM, Foggin C, Hove T, de Garine-Wichatitsky M (2013) Relationship between burden of infection in ungulate populations and wildlife/livestock interfaces. Epidemiol Infect 141:1522-1535

92. Carrasco-Garcia R, Barasona JA, Gortazar C, Montoro V, Sanchez-Vizcaino JM, Vicente J (2016) Wildlife and livestock use of extensive farm resources in South Central Spain: implications for disease transmission. Eur J Wildl Res 62:65-78

93. Carrique-Mas JJ, Medley GF, Green LE (2008) Risks for bovine tuberculosis in British cattle farms restocked after the foot and mouth disease epidemic of 2001. Prev Vet Med 84:85-93

94. Carter SP, Chambers MA, Rushton SP, Shirley MDF, Schuchert P, Pietravalle S, Murray A, Rogers F, Gettinby G, Smith GC, Delahay RJ, Hewinson RG, McDonald RA (2012) BCG vaccination reduces risk of tuberculosis infection in vaccinated badgers and unvaccinated badger cubs. PLoS One 7:8

95. Castillo L, Fernandez-Llario P, Mateos C, Carranza J, Benitez-Medina JM, Garcia-Jimenez W, Bermejo-Martin F, de Mendoza JH (2011) Management practices and their association with *Mycobacterium tuberculosis complex* prevalence in red deer populations in Southwestern Spain. Prev Vet Med 98:58-63

96. Cezar RDD, Lucena-Silva N, Batista AF, Borges JD, de Oliveira PRF, Lucio EC, Arruda-Lima M, Santana VLD, Pinheiro JW (2016) Molecular detection of *Mycobacterium bovis* in cattle herds of the state of Pernambuco, Brazil. BMC Vet Res 12:6

97. Chambers MA, Rogers F, Delahay RJ, Lesellier S, Ashford R, Dalley D, Gowtage S, Dave D, Palmer S, Brewer J, Crawshaw T, Clifton-Hadley R, Carter S, Cheeseman C, Hanks C, Murray A, Palphramand K, Pietravalle S, Smith GC, Tomlinson A, Walker NJ, Wilson GJ, Corner LA, Rushton SP, Shirley MD, Gettinby G, McDonald RA, Hewinson RG (2011) Bacillus Calmette-Guerin vaccination reduces the severity and progression of tuberculosis in badgers. Proc Biol Sci 278:1913-1920

98. Che'Amat A, Armenteros JA, Gonzalez-Barrio D, Lima JF, Diez-Delgado I, Barasona JA, Romero B, Lyashchenko KP, Ortiz JA, Gortazar C (2016) Is targeted removal a suitable means for tuberculosis control in wild boar? Prev Vet Med 135:132-135

99. Cheeseman CL, Jones GW, Gallagher J, Mallinson PJ (1981) The population-structure, density and prevalence of tuberculosis (*Mycobacterium bovis*) in badgers (*Meles meles*) from 4 areas in Southwest England. J Appl Ecol 18:795-804

100. Ciaravino G, Garcia-Saenz A, Cabras S, Allepuz A, Casal J, Garcia-Bocanegra I, De Koeijer A, Gubbins S, Saez JL, Cano-Terriza D, Napp S (2018) Assessing the variability in transmission of bovine tuberculosis within Spanish cattle herds. Epidemics 23:110-120

101. Ciaravino G, Laranjo-Gonzalez M, Casal J, Saez-Llorente JL, Allepuz A (2021) Most likely causes of infection and risk factors for tuberculosis in Spanish cattle herds. Vet Rec 189:11

102. Clarke A, Byrne AW, Maher J, Ryan E, Farrell F, McSweeney C, Barrett D (2022) Engaging with farmers to explore correlates of bovine tuberculosis risk in an internationally important heritage landscape: The Burren, in the West of Ireland. Front Vet Sci 9:9

103. Cleaveland S, Shaw DJ, Mfinanga SG, Shirima G, Kazwala RR, Eblate E, Sharp M (2007) *Mycobacterium bovis* in rural Tanzania: Risk factors for infection in human and cattle populations. Tuberculosis 87:30-43

104. Clegg TA, Blake M, Healy R, Good M, Higgins IM, More SJ (2013) The impact of animal introductions during herd restrictions on future herd-level bovine tuberculosis risk. Prev Vet Med 109:246-257

105. Clegg TA, Good M, Hayes M, Duignan A, McGrath G, More SJ (2018) Trends and predictors of large tuberculosis episodes in cattle herds in Ireland. Front Vet Sci 5:12

106. Clegg TA, Good M, More SJ (2016) Risk factors for cattle presenting with a confirmed bTB lesion at slaughter, from herds with no evidence of within-herd transmission. Prev Vet Med 126:111-120

107. Cliftonhadley RS, Wilesmith JW, Richards MS, Upton P, Johnston S (1995) The occurrence of *Mycobacterium bovis* infection in cattle in and around an area subject to extensive badger (*Meles meles*) control. Epidemiol Infect 114:179-193

108. Coleman JD, Coleman MC, Warburton B (2006) Trends in the incidence of tuberculosis in possums and livestock, associated with differing control intensities applied to possum populations. New Zealand Vet J 54:52-60

109. Coleman JD, Cooke MM, Jackson R, Webster R (1999) Temporal patterns in bovine tuberculosis in a brushtail possum population contiguous with infected cattle in the Ahaura Valley, Westland. New Zealand Vet J 47:119-124

110. Coleman JD, Jackson R, Cooke MM, Grueber L (1994) Prevalence and spatial-distribution of bovine tuberculosis in brushtail possums on a forest-scrub margin. New Zealand Vet J 42:128-132

111. Conlan AJK, McKinley TJ, Karolemeas K, Pollock EB, Goodchild AV, Mitchell AP, Birch CPD, Clifton-Hadley RS, Wood JLN (2012) Estimating the hidden burden of bovine tuberculosis in Great Britain. PLoS Comput Biol 8:14

112. Cook AJC, Tuchili LM, Buve A, Foster SD, GodfreyFaussett P, Pandey GS, McAdam K (1996) Human and bovine tuberculosis in the Monze District of Zambia - A cross-sectional study. British Vet J 152:37-46

113. Corn JL, Nettles VF (2001) Health protocol for translocation of free-ranging elk. J Wildl Dis 37:413-426

114. Corner LAL, Clegg TA, More SJ, Williarns DH, O'Boyle I, Costello E, Sleeman DP, Griffin JM (2008) The effect of varying levels of population control on the prevalence of tuberculosis in badgers in Ireland. Res J Vet Sci 85:238-249

115. Corner LAL, Norton S, Buddle BM, Morris RS (2002) The efficacy of bacille Calmette-Guerin vaccine in wild brushtail possums (*Trichosurus vulpecula*). Res J Vet Sci 73:145-152

116. Corner LAL, Pfeiffer DU, de Lisle GW, Morris RS, Buddle BM (2002) Natural transmission of *Mycobacterium bovis* infection in captive brushtail possums (*Trichosurus vulpecula*). New Zealand Vet J 50:154-162

117. Corner LAL, Pfeiffer DU, Morris RS (2003) Social-network analysis of *Mycobacterium bovis* transmission among captive brushtail possums (*Trichosurus vulpecula*). Prev Vet Med 59:147-167

118. Corner LAL, Stevenson MA, Collins DM, Morris RS (2003) The re-emergence of *Mycobacterium bovis* infection in brushtail possums (*Trichosurus vulpecula*) after localised possum eradication. New Zealand Vet J 51:73-80

119. Cosgrove MK, Campa H, Ramsey DSL, Schmitt SM, O'Brien DJ (2012) Modeling Vaccination and targeted removal of white-tailed deer in Michigan for bovine tuberculosis control. Wildl Soc Bull 36:676-684

120. Cosgrove MK, Campa H, Schmitt SM, Marks DR, Wilson AS, O'Brien DJ (2012) Live-trapping and bovine tuberculosis testing of free-ranging white-tailed deer for targeted removal. Wildl Res 39:104-111

121. Costello E, Doherty ML, Monaghan ML, Quigley FC, O'Reilly PF (1998) A study of cattle-to-cattle transmission of *Mycobacterium bovis* infection. Vet J 155:245-250

122. Cowie CE, Beck BB, Gortazar C, Vicente J, Hutchings MR, Moran D, White PCL (2014) Risk factors for the detected presence of *Mycobacterium bovis* in cattle in south central Spain. Eur J Wildl Res 60:113-123

123. Cowie CE, Hutchings MR, Barasona JA, Gortazar C, Vicente J, White PCL (2016) Interactions between four species in a complex wildlife: livestock disease community: implications for *Mycobacterium bovis* maintenance and transmission. Eur J Wildl Res 62:51-64

124. Cox DR, Donnelly CA, Bourne FJ, Gettinby G, McInerney JP, Morrison WI, Woodroffe R (2005) Simple model for tuberculosis in cattle and badgers. PNAS USA 102:17588-17593

125. Crispell J, Benton CH, Balaz D, De Maio N, Ahkmetova A, Allen A, Biek R, Presho EL, Dale J, Hewinson G, Lycett SJ, Nunez-Garcia J, Skuce RA, Trewby H, Wilson DJ, Zadoks RN, Delahay RJ, Kao RR (2019) Combining genomics and epidemiology to analyse bi-directional transmission of *Mycobacterium bovis* in a multi-host system. Elife 8:36

126. Crispell J, Zadoks RN, Harris SR, Paterson B, Collins DM, de-Lisle GW, Livingstone P, Neill MA, Biek R, Lycett SJ, Kao RR, Price-Carter M (2017) Using whole genome sequencing to investigate transmission in a multi-host system: bovine tuberculosis in New Zealand. BMC Genom 18:12

127. Cross PC, Getz WM (2006) Assessing vaccination as a control strategy in an ongoing epidemic: Bovine tuberculosis in African buffalo. Ecol Modell 196:494-504

128. Cross PC, Lloyd-Smith JO, Bowers JA, Hay CT, Hofmeyr M, Getz WM (2004) Integrating association data and disease dynamics in a social ungulate: bovine tuberculosis in African buffalo in the Kruger National Park. Ann Zool Fenn 41:879-892

129. Cvetkovikj I, Mrenoshki S, Krstevski K, Djadjovski I, Angjelovski B, Popova Z, Janevski A, Dodovski A, Cvetkovikj A (2017) Bovine tuberculosis in the Republic of Macedonia: postmortem, microbiological and molecular study in slaughtered reactor cattle. Macedonian Vet Rev 40:43-52

130. Day TD, O'Connor CE, Waas JR (2000) Den sharing behaviour of captive brushtail possums (*Trichosurus vulpecula*). New Zealand J Zool 27:183-187

131. de la Cruz ML, Perez A, Bezos J, Pages E, Casal C, Carpintero J, Romero B, Dominguez L, Barker CM, Diaz R, Alvarez J (2014) Spatial dynamics of bovine tuberculosis in the autonomous community of Madrid, Spain (2010-2012). PLoS One 9:16

132. de Mendoza JH, Parra A, Tato A, Alonso JM, Rey JM, Pena J, Garcia-Sanchez A, Larrasa J, Teixido J, Manzano G, Cerrato R, Pereira G, Fernández-Llario P, Hermoso de Mendoza M (2006) Bovine tuberculosis in wild boar (*Sus scrofa*), red deer (*Cervus elaphus*) and cattle (*Bos taurus*) in a Mediterranean ecosystem (1992-2004). Prev Vet Med 74:239-247

133. Dejene SW, Heitkonig IMA, Prins HHT, Lemma FA, Mekonnen DA, Alemu ZE, Kelkay TZ, de Boer WF (2016) Risk Factors for bovine tuberculosis (bTB) in cattle in Ethiopia. PLoS One 11:16

134. Delahay RJ, Langton S, Smith GC, Clifton-Hadley RS, Cheeseman CL (2000) The spatio-temporal distribution of *Mycobacterium bovis* (bovine tuberculosis) infection in a high-density badger population. J Anim Ecol 69:428-441

135. Delahay RJ, Smith GC, Barlow AM, Walker N, Harris A, Clifton-Hadley RS, Cheeseman CL (2007) Bovine tuberculosis infection in wild mammals in the South-West region of England: A survey of prevalence and a semi-quantitative assessment of the relative risks to cattle. Vet J 173:287-301

136. Delahay RJ, Walker N, Smith GS, Wilkinson D, Clifton-Hadley RS, Cheeseman CL, Tomlinson AJ, Chambers MA (2013) Long-term temporal trends and estimated transmission rates for *Mycobacterium bovis* infection in an undisturbed high-density badger (*Meles meles*) population. Epidemiol Infect 141:1445-1456

137. Demelash B, Inangolet F, Oloya J, Asseged B, Badaso M, Yilkal A, Skjerve E (2009) Prevalence of bovine tuberculosis in ethiopian slaughter cattle based on post-mortem examination. Trop Anim Health Prod 41:755-765

138. Denny GO, Wilesmith JW (1999) Bovine tuberculosis in Northern Ireland: a case-control study of herd risk factors. Vet Rec 144:305-310

139. Dias RA, Ulloa-Stanojlovic FM, Belchior APC, Ferreira RD, Goncalves RC, de Aguiar R, Sousa PD, Santos AMA, Amaku M, Ferreira F, Telles EO, Grisi-Filho JHH, Gonçalves VSP, Heinemann MB, Ferreira Neto JS (2016) Prevalence and risk factors for bovine tuberculosis in the state of Sao Paulo, Brazil. Semin Cienc Agrar 37:3673-3683

140. Diez-Delgado I, Sevilla IA, Romero B, Tanner E, Barasona JA, White AR, Lurz PWW, Boots M, de la Fuente J, Dominguez L, Vicente J, Garrido JM, Juste RA, Aranaz A, Gortazar C (2018) Impact of piglet oral vaccination against tuberculosis in endemic free-ranging wild boar populations. Prev Vet Med 155:11-20

141. Dinka H, Duressa A (2011) Prevalence of bovine tuberculosis in Arsi Zones of Oromia, Ethiopia. Afr J Agric Res 6:3853-3858

142. Djafar ZR, Benazi N, Bounab S, Sayhi M, Diouani MF, Benia F (2020) Distribution of seroprevalence and risk factors for bovine tuberculosis in east Algeria. Prev Vet Med 183:8

143. Dommergues L, Rautureau S, Petit E, Dufour B (2012) Network of contacts between cattle herds in a french area affected by bovine tuberculosis in 2010. Transbound Emerg Dis 59:292-302

144. Donnelly CA, Bento AI, Goodchild AV, Downs SH (2015) Exploration of the power of routine surveillance data to assess the impacts of industry-led badger culling on bovine tuberculosis incidence in cattle herds. Vet Rec 177:4

145. Donnelly CA, Wei G, Johnston WT, Cox DR, Woodroffe R, Bourne FJ, Cheeseman CL, Clifton-Hadley RS, Gettinby G, Gilks P, Jenkins HE, Le Fevre AM, McInerney JP, Morrison WI (2007) Impacts of widespread badger culling on cattle tuberculosis: concluding analyses from a large-scale field trial. IJID 11:300-308

146. Donnelly CA, Woodroffe R, Cox DR, Bourne FJ, Cheeseman CL, Clifton-Hadley RS, Wei G, Gettinby G, Gilks P, Jenkins H, Johnston WT, Le Fevre AM, McInerney JP, Morrison WI (2006) Positive and negative effects of widespread badger culling on tuberculosis in cattle. Nature 439:843-846

147. Donnelly CA, Woodroffe R, Cox DR, Bourne J, Gettinby G, Le Fevre AM, McInerney JP, Morrison WI (2003) Impact of localized badger culling on tuberculosis incidence in British cattle. Nature 426:834-837

148. Downs SH, Ashfield S, Arnold M, Roberts T, Prosser A, Robertson A, Frost S, Harris K, Avigad R, Smith GC (2022) Detection of a local *Mycobacterium bovis* reservoir using cattle surveillance data. Transbound Emerg Dis 69:e104-e118

149. Doyle LP, Courcier EA, Gordon AW, O'Hagan MJH, Stegeman JA, Menzies FD (2017) Bovine tuberculosis in Northern Ireland: quantification of the population disease-level effect from cattle leaving herds detected as a source of infection. Epidemiol Infect 145:3505-3515

150. Drewe JA, O'Connor HM, Weber N, McDonald RA, Delahay RJ (2013) Patterns of direct and indirect contact between cattle and badgers naturally infected with tuberculosis. Epidemiol Infect 141:1467-1475

151. Duault H, Michelet L, Boschiroli ML, Durand B, Canini L (2022) A Bayesian evolutionary model towards understanding wildlife contribution to F4-family *Mycobacterium bovis* transmission in the South-West of France. BMC Vet Res 53:12

152. Echeverria G, Ron L, Leon AM, Espinosa W, Benitez-Ortiz W, Proano-Perez F (2014) Prevalence of bovine tuberculosis in slaughtered cattle identified by nested-PCR in abattoirs from two dairy areas of Ecuador. Trop Anim Health Prod 46:1015-1022

153. Egbe NF, Muwonge A, Ndip L, Kelly RF, Sander M, Tanya V, Ngwa VN, Handel IG, Novak A, Ngandalo R, Mazeri S, Morgan KL, Asuquo A, Bronsvoort BM (2016) Abattoir-based estimates of mycobacterial infections in Cameroon. Sci Rep 6:14

154. Elias K, Hussein D, Asseged B, Wondwossen T, Gebeyehu M (2008) Status of bovine tuberculosis in Addis Ababa dairy farms. OMSA 27:915-923

155. Enriquez-Cruz C, Cruz-Hernandez NI, Zertuche-Rodriguez JL, Uriegas-Garcia JL, Toscano-Ruiz JE, Flores-Gutierrez GH (2010) Epidemiology of bovine tuberculosis in Mexico, bordering the United States, at establishment of controlling strategies. Arq Bras Med Vet 62:1029-1035

156. Escarcega DAV, Razo CAP, Ruiz SG, Gallegos SLS, Suazo FM, Alarcon GJC (2020) Analysis of bovine tuberculosis transmission in Jalisco, Mexico through whole-genome sequencing. J Vet Res 64:51-61

157. Esmaeilzadeh N, Bahonar A, Foroushani AR, Nasehi M, Amiri K, Hadjzadeh MAR (2022) Temporal trends and prediction of bovine tuberculosis: a time series analysis in the North-East of Iran. Iran J Vet Res 23:12-17

158. Fenichel EP, Horan RD (2007) Gender-Based harvesting in wildlife disease management. AJAE 89:904-920

159. Fenichel EP, Horan RD (2007) Jointly-determined ecological thresholds and economic trade-offs in wildlife disease management. NRM 20:511-547

160. Fenwick NID (2012) Modelled impacts of badger culling on cattle TB in a real area with geographic boundaries. Vet Rec 170:177

161. Fetene T, Kebede N (2009) Bovine tuberculosis of cattle in three districts of northwestern Ethiopia. Trop Anim Health Prod 41:273-277

162. Fielding HR, McKinley TJ, Delahay RJ, Silk MJ, McDonald RA (2020) Effects of trading networks on the risk of bovine tuberculosis incidents on cattle farms in Great Britain. R Soc Open Sci 7:11

163. Fielding HR, McKinley TJ, Silk MJ, Delahay RJ, McDonald RA (2019) Contact chains of cattle farms in Great Britain. R Soc Open Sci 6:16

164. Fielding HR, Silk MJ, McKinley TJ, Delahay RJ, Wilson-Aggarwal JK, Gauvin L, Ozella L, Cattuto C, McDonald RA (2021) Spatial and temporal variation in proximity networks of commercial dairy cattle in Great Britain. Prev Vet Med 194:11

165. Firdessa R, Tschopp R, Wubete A, Sombo M, Hailu E, Erenso G, Kiros T, Yamuah L, Vordermeier M, Hewinson RG, Young D, Gordon SV, Sahile M, Aseffa A, Berg S (2012) High prevalence of bovine tuberculosis in dairy cattle in Central Ethiopia: Implications for the Dairy Industry and Public Health. PLoS One 7:12

166. Foddai A, Nielson LR, Krogh K, Alban L (2015) Assessment of the probability of introduction of bovine tuberculosis to Danish cattle farms via imports of live cattle from abroad and immigrant workers. Prev Vet Med 122:306-317

167. Fulford GR, Roberts MG, Heesterbeek JAP (2002) The metapopulation dynamics of an infectious disease: Tuberculosis in possums. Theor Popul Biol 61:15-29

168. Galvis JOA, Grisi JHH, da Costa D, Said A, Amaku M, Dias RA, Ferreira F, Goncalves VSP, Heinemann MB, Telles EO, Neto JSF (2016) Epidemiologic characterization of bovine tuberculosis in the state of Espirito Santo, Brazil. Semin Cienc Agrar 37:3567-3578

169. Garcia-Bocanegra I, de Val BP, Arenas-Montes A, Paniagua J, Boadella M, Gortazar C, Arenas A (2012) Seroprevalence and risk factors associated to *Mycobacterium bovis* in wild artiodactyl species from Southern Spain, 2006-2010. PLoS One 7:8

170. Garcia-Jimenez WL, Fernandez-Llario P, Benitez-Medina JM, Cerrato R, Cuesta J, Garcia-Sanchez A, Goncalves P, Martinez R, Risco D, Salguero FJ, Serrano E, Gómez L, Hermoso-de-Mendoza J (2013) Reducing Eurasian wild boar (*Sus scrofa*) population density as a measure for bovine tuberculosis control: Effects in wild boar and a sympatric fallow deer (*Dama dama*) population in Central Spain. Prev Vet Med 110:435-446

171. Garnett BT, Delahay RJ, Roper TJ (2002) Use of cattle farm resources by badgers (Meles meles) and risk of bovine tuberculosis (*Mycobacterium bovis*) transmission to cattle. Proc Biol Sci 269:1487-1491

172. Gates MC, Volkova VV, Woolhouse MEJ (2013) Risk factors for bovine tuberculosis in low incidence regions related to the movements of cattle. BMC Vet Res 9:16

173. Gates MC, Woolhouse MEJ (2014) Suboptimal herd performance amplifies the spread of infectious disease in the cattle industry. PLoS One 9:10

174. Gaughran A, Mullen E, MacWhite T, Maher P, Kelly DJ, Kelly R, Good M, Marples NM (2021) Badger territoriality maintained despite disturbance of major road construction. PLoS One 16:20

175. Georgaki A, Bishop H, Gordon A, Doyle L, O'Hagan M, Courcier E, Menzies F (2022) Evaluating the risk of bovine tuberculosis posed by standard inconclusive reactors identified at backward-traced herd tests in Northern Ireland that disclosed no reactors. Res J Vet Sci 145:205-212

176. Ghebremariam MK, Michel AL, Nielen M, Vernooij JCM, Rutten V (2018) Farm-level risk factors associated with bovine tuberculosis in the dairy sector in Eritrea. Transbound Emerg Dis 65:105-113

177. Ghebremariam MK, Michel AL, Vernooij JCM, Nielen M, Rutten V (2018) Prevalence of bovine tuberculosis in cattle, goats, and camels of traditional livestock raising communities in Eritrea. BMC Vet Res 14:13

178. Ghebremariam MK, Rutten V, Vernooij JCM, Uqbazghi K, Tesfaalem T, Butsuamlak T, Idris AM, Nielen M, Michel AL (2016) Prevalence and risk factors of bovine tuberculosis in dairy cattle in Eritrea. BMC Vet Res 12:7

179. Gilbert M, Mitchell A, Bourn D, Mawdsley J, Cliton-Hadley R, Wint W (2005) Cattle movements and bovine tuberculosis in Great Britain. Nature 435:491-496

180. Gong QL, Chen Y, Tian T, Wen XB, Li D, Song YH, Wang Q, Du R, Zhang XX (2021) Prevalence of bovine tuberculosis in dairy cattle in China during 2010-2019: A systematic review and meta-analysis. PLoS Negl Trop Dis 15:20

181. Good M, Clegg TA, Duignan A, More SJ (2011) Impact of the national full herd depopulation policy on the recurrence of bovine tuberculosis in Irish herds, 2003 to 2005. Vet Rec 169:581-551

182. Gopal R, Goodchild A, Hewinson G, Domenech R, Clifton-Hadley R (2006) Introduction of bovine tuberculosis to north-east England by bought-in cattle. Vet Rec 159:265-271

183. Gormley AM, Anderson DP, Nugent G (2018) Cost-based optimization of the stopping threshold for local disease surveillance during progressive eradication of tuberculosis from New Zealand wildlife. Transbound Emerg Dis 65:186-196

184. Gormley AM, Holland EP, Barron MC, Anderson DP, Nugent G (2016) A modelling framework for predicting the optimal balance between control and surveillance effort in the local eradication of tuberculosis in New Zealand wildlife. Prev Vet Med 125:10-18

185. Gormley E, Bhuachalla DN, Fitzsimons T, O'Keeffe J, McGrath G, Madden JM, Fogarty N, Kenny K, Messam LLM, Murphy D, Corner LAL (2022) Protective immunity against tuberculosis in a free-living badger population vaccinated orally with *Mycobacterium bovis* Bacille Calmette-Guerin. Transbound Emerg Dis 69:e10-e19

186. Gormley E, Bhuachalla DN, O'Keeffe J, Murphy D, Aldwell FE, Fitzsimons T, Stanley P, Tratalos JA, McGrath G, Fogarty N, Kenny K, More SJ, Messam LL, Corner LA (2017) Oral vaccination of free-living badgers (*Meles meles*) with Bacille Calmette Guerin (BCG) Vaccine confers protection against tuberculosis. PLoS One 12:16

187. Gortazar C, Beltran-Beck B, Garrido JM, Aranaz A, Sevilla IA, Boadella M, Lyashchenko KP, Galindo RC, Montoro V, Dominguez L, Juste R, de la Fuente J (2014) Oral re-vaccination of Eurasian wild boar with *Mycobacterium bovis* BCG yields a strong protective response against challenge with a field strain. BMC Vet Res 10:7

188. Gortazar C, Fernandez-Calle LM, Collazos-Martinez JA, Minguez-Gonzalez O, Acevedo P (2017) Animal tuberculosis maintenance at low abundance of suitable wildlife reservoir hosts: A case study in northern Spain. Prev Vet Med 146:150-157

189. Gortazar C, Torres MJ, Vicente J, Acevedo P, Reglero M, de la Fuente J, Negro JJ, Aznar-Martin J (2008) Bovine tuberculosis in donana biosphere reserve: the role of wild ungulates as disease reservoirs in the last iberian lynx strongholds. PLoS One 3:8

190. Graham J, Smith GC, Delahay RJ, Bailey T, McDonald RA, Hodgson D (2013) Multi-state modelling reveals sex-dependent transmission, progression and severity of tuberculosis in wild badgers. Epidemiol Infect 141:1429-1436

191. Green DM, Kiss IZ, Mitchell AP, Kao RR (2008) Estimates for local and movement-based transmission of bovine tuberculosis in British cattle. Proc Biol Sci 275:1001-1005

192. Green LE, Carrique-Mas JJ, Mason SA, Medley GF (2012) Patterns of delayed detection and persistence of bovine tuberculosis in confirmed and unconfirmed herd breakdowns in cattle and cattle herds in Great Britain. Prev Vet Med 106:266-274

193. Green LE, Cornell SJ (2005) Investigations of cattle herd breakdowns with bovine tuberculosis in four counties of England and Wales using VETNET data. Prev Vet Med 70:293-311

194. Green WQ, Coleman JD (1986) Movement of possums (*Trichosurus vulpecula*) between forest and pasture in westland, New-Zealand - implications for bovine tuberculosis transmission. New Zealand J Ecol 9:57-69

195. Greenman JV, Hoyle AS (2008) Exclusion of generalist pathogens in multihost communities. Am Nat 172:576-584

196. Griffin JM, Martin SW, Thorburn MA, Eves JA, Hammond RF (1996) A case-control study on the association of selected risk factors with the occurrence of bovine tuberculosis in the Republic of Ireland. Prev Vet Med 27:217-229

197. Griffin JM, More SJ, Clegg TA, Collins JD, O'Boyle I, Williams DH, Kelly GE, Costello E, Sleeman DP, O'Shea F, Duggan M, Murphy J, Lavin DP (2005) Tuberculosis in cattle: the results of the four-area project. Irish Vet J 58:629-636

198. Griffin JM, Williams DH, Kelly GE, Clegg TA, O'Boyle I, Collins JD, More SJ (2005) The impact of badger removal on the control of tuberculosis in cattle herds in Ireland. Prev Vet Med 67:237-266

199. Guedes IB, Bottene IFN, Monteiro L, Leal JM, Heinemann MB, Amaku M, Grisi JHH, Dias RA, Ferreira F, Telles EO, Gonçalves VSP, Ferreira Neto JS (2016) Prevalence and risk factors for bovine tuberculosis in the State of Mato Grosso do Sul, Brazil. Semin Cienc Agrar 37:3579-3588

200. Gumi B, Schelling E, Firdessa R, Aseffa A, Tschopp R, Yamuah L, Young D, Zinsstag J (2011) Prevalence of bovine tuberculosis in pastoral cattle herds in the Oromia region, southern Ethiopia. Trop Anim Health Prod 43:1081-1087

201. Guta S, Casal J, Garcia-Saenz A, Saez JL, Pacios A, Garcia P, Napp S, Allepuz A (2014) Risk factors for bovine tuberculosis persistence in beef herds of Southern and Central Spain. Prev Vet Med 115:173-180

202. Guta S, Casal J, Napp S, Saez JL, Garcia-Saenz A, de Val BP, Romero B, Alvarez J, Allepuz A (2014) Epidemiological investigation of bovine tuberculosis herd breakdowns in Spain 2009/2011. PLoS One 9:12

203. Habitu T, Areda D, Muwonge A, Tessema GT, Skjerve E, Gebrehiwot T (2019) Prevalence and risk factors analysis of bovine tuberculosis in cattle raised in mixed crop-livestock farming system in Tigray region, Ethiopia. Transbound Emerg Dis 66:488-496

204. Ham C, Donnelly CA, Astley KL, Jackson SYB, Woodroffe R (2019) Effect of culling on individual badger *Meles meles* behaviour: Potential implications for bovine tuberculosis transmission. J Appl Ecol 56:2390-2399

205. Hardstaff JL, Bulling MT, Marion G, Hutchings MR, White PCL (2012) Impact of external sources of infection on the dynamics of bovine tuberculosis in modelled badger populations. BMC Vet Res 8:10

206. Hardstaff JL, Bulling MT, Marion G, Hutchings MR, White PCL (2013) Modelling the impact of vaccination on tuberculosis in badgers. Epidemiol Infect 141:1417-1427

207. Hone J, Donnelly CA (2008) Evaluating evidence of association of bovine tuberculosis in cattle and badgers. J Appl Ecol 45:1660-1666

208. Horan RD, Wolf CA (2005) The economics of managing infectious wildlife disease. AJAE 87:537-551

209. Horan RD, Wolf CA, Fenichel EP, Mathews KH (2008) Joint management of wildlife and livestock disease. ERE 41:47-70

210. Houtsma E, Clegg TA, Good M, More SJ (2018) Further improvement in the control of bovine tuberculosis recurrence in Ireland. Vet Rec 183:622

211. Huang ZYX, de Boer WF, van Langevelde F, Xu C, Ben Jebara K, Berlingieri F, Prins HHT (2013) Dilution effect in bovine tuberculosis: risk factors for regional disease occurrence in Africa. Proc Biol Sci 280:7

212. Huang ZYX, Xu C, van Langevelde F, Prins HHT, ben Jebara K, de Boer WF (2014) Dilution effect and identity effect by wildlife in the persistence and recurrence of bovine tuberculosis. Parasitology 141:981-987

213. Humblet MF, Gilbert M, Govaerts M, Fauville-Dufaux M, Walravens K, Saegerman C (2010) New assessment of bovine tuberculosis risk factors in Belgium based on nationwide molecular epidemiology. J Clin Microbiol 48:2802-2808

214. Hutchings MR, Harris S (1999) Quantifying the risks of TB infection to cattle posed by badger excreta. Epidemiol Infect 122:167-173

215. Ibrahim S, Agada CA, Umoh JU, Ajogi I, Farouk UM, Cadmus SIB (2010) Prevalence of bovine tuberculosis in Jigawa State, northwestern Nigeria. Trop Anim Health Prod 42:1333-1335

216. Islam MN, Khan MK, Khan MFR, Kostoulas P, Rahman A, Alam MM (2021) Risk factors and true prevalence of bovine tuberculosis in Bangladesh. PLoS One 16:15

217. Islam SKS, Rumi TB, Kabir SML, van der Zanden AGM, Kapur V, Rahman A, Ward MP, Bakker D, Ross AG, Rahim Z (2020) Bovine tuberculosis prevalence and risk factors in selected districts of Bangladesh. PLoS One 15:18

218. Jajere SM, Atsanda NN, Bitrus AA, Hamisu TM, Goni MD (2018) Occurrence of bovine tuberculosis among cattle herds from nomadic peri-urban settlements and cattle slaughtered at the municipal abattoir of Bauchi, NorthEastern Nigeria. JAVAR 5:53-59

219. Jajere SM, Atsanda NN, Bitrus AA, Hamisu TM, Goni MD (2018) A retrospective study of bovine tuberculosis at the municipal abattoir of Bauchi State, Northeastern Nigeria. Vet World 11:598-605

220. Javed MT, Irfan M, Ali I, Farooqi FA, Wasiq M, Cagiola M (2011) Risk factors identified associated with tuberculosis in cattle at 11 livestock experiment stations of Punjab Pakistan. Acta Tropica 117:109-113

221. Javed MT, Wasiq M, Farooqi FA, Shahid AL, Kausar R, Cagiola M (2013) Certain risk factors associated with positive SCCIT test for tuberculosis in cattle at two cities in Pakistan. Asian Biomed 7:267-274

222. Jenkins HE, Cox DR, Delahay RJ (2012) Direction of association between bite wounds and *Mycobacterium bovis* infection in badgers: implications for transmission. PLoS One 7:7

223. Jenkins HE, Woodroffe R, Donnelly CA (2008) The effects of annual widespread badger culls on cattle tuberculosis following the cessation of culling. IJID 12:457-465

224. Jenkins HE, Woodroffe R, Donnelly CA (2010) The duration of the effects of repeated widespread badger culling on cattle tuberculosis following the cessation of culling. PLoS One 5:7

225. Jenkins HE, Woodroffe R, Donnelly CA, Cox DR, Johnston WT, Bourne FJ, Cheeseman CL, Clifton-Hadley RS, Gettinby G, Gilks P, Hewinson RG, Mcinerney JP Morrison WI  (2007) Effects of culling on spatial associations of *Mycobacterium bovis* infections in badgers and cattle. J Appl Ecol 44:897-908

226. Ji WH, Sarre SD, Craig JL, Clout MN (2003) Denning behavior of common brushtail possums in populations recovering from density reduction. J Mammal 84:1059-1067

227. Jiwa SFH, Kazwala RR, Aboud AAO, Kalaye WJ (1997) Bovine tuberculosis in the Lake Victoria Zone of Tanzania and its possible consequences for human health in the HIV/AIDS era. Vet Res Commun 21:533-539

228. Johnston WT, Gettinby G, Cox DR, Donnelly CA, Bourne J, Clifton-Hadley R, Le Fevre AM, McInerney JP, Mitchell A, Morrison WI, Woodroffe R (2005) Herd-level risk factors associated with tuberculosis breakdowns among cattle herds in England before the 2001 foot-and-mouth disease epidemic. Biol Lett 1:53-56

229. Johnston WT, Vial F, Gettinby G, Bourne FJ, Clifton-Hadley RS, Cox DR, Crea P, Donnelly CA, McInerney JP, Mitchell AP, Morrison WI, Woodroffe R (2011) Herd-level risk factors of bovine tuberculosis in England and Wales after the 2001 foot-and-mouth disease epidemic. IJID 15:e833-e840

230. Judge J, McDonald RA, Walker N, Delahay RJ (2011) Effectiveness of biosecurity measures in preventing badger visits to farm buildings. PLoS One 6:8

231. Kaneene JB, Bruning-Fann CS, Granger LM, Miller RA, Porter-Spalding BA (2002) Environmental and farm management factors associated with tuberculosis on cattle farms in northeastern Michigan. JAVMA 221:837-842

232. Kao RR, Roberts MG, Ryan TJ (1997) A model of bovine tuberculosis control in domesticated cattle heeds. Proc Biol Sci 264:1069-1076

233. Karolemeas K, Donnelly CA, Conlan AJK, Mitchell AP, Clifton-Hadley RS, Upton P, Wood JLN, McKinley TJ (2012) The effect of badger culling on breakdown prolongation and recurrence of bovine tuberculosis in cattle herds in Great Britain. PLoS One 7:8

234. Karolemeas K, McKinley TJ, Clifton-Hadley RS, Goodchild AV, Mitchell A, Johnston WT, Conlan AJK, Donnelly CA, Wood JLN (2010) Predicting prolonged bovine tuberculosis breakdowns in Great Britain as an aid to control. Prev Vet Med 97:183-190

235. Karolemeas K, McKinley TJ, Clifton-Hadley RS, Goodchild AV, Mitchell A, Johnston WT, Conlan AJK, Donnelly CA, Wood JLN (2011) Recurrence of bovine tuberculosis breakdowns in Great Britain: Risk factors and prediction. Prev Vet Med 102:22-29

236. Katale BZ, Fyumagwa RD, Mjingo EE, Sayalel K, Batamuzi EK, Matee MI, Keyyu JD, Muumba J, Mdaki M, Mbugi EV, Rweyemamu MM, Mpanduji DG (2017) Screening for bovine tuberculosis in african buffalo (*Syncerus caffer*) in Ngorongoro conservation area, Northern Tanzania: implications for public health. J Wildl Dis 53:711-717

237. Katale BZ, Mbugi EV, Karimuribo ED, Keyyu JD, Kendall S, Kibiki GS, Godfrey-Faussett P, Michel AL, Kazwala RR, van Helden P, Matee MI (2013) Prevalence and risk factors for infection of bovine tuberculosis in indigenous cattle in the Serengeti ecosystem, Tanzania. BMC Vet Res 9:11

238. Kazoora HB, Majalija S, Kiwanuka N, Kaneene JB (2014) Prevalence of *Mycobacterium bovis* skin positivity and associated risk factors in cattle from Western Uganda. Trop Anim Health Prod 46:1383-1390

239. Kazwala RR, Kambarage DM, Daborn CJ, Nyange J, Jiwa SFH, Sharp JM (2001) Risk factors associated with the occurrence of bovine tuberculosis in cattle in the Southern Highlands of Tanzania. Vet Res Commun 25:609-614

240. Kean JM, Barlow ND, Hickling GJ (1999) Evaluating potential sources of bovine tuberculosis infection in a New Zealand cattle herd. New Zealand J Agr Res 42:101-106

241. Kelly DJ, Mullen E, Good M (2021) Bovine Tuberculosis: the emergence of a new wildlife maintenance host in Ireland. Front Vet Sci 8:7

242. Kelly GE (2011) Anisotropic spatial clustering of TB in cattle - the implications for control policy. Procedia Environ Sci 3: 111-116

243. Kelly GE, Condon J, More SJ, Dolan L, Higgins I, Eves J (2008) A long-term observational study of the impact of badger removal on herd restrictions due to bovine TB in the Irish midlands during 1989-2004. Epidemiol Infect 136:1362-1373

244. Kelly GE, McGrath GE, More SJ (2010) Estimating the extent of spatial association of *Mycobacterium bovis* infection in badgers in Ireland. Epidemiol Infect 138:270-279

245. Kelly GE, More SJ (2011) Spatial clustering of TB-infected cattle herds prior to and following proactive badger removal. Epidemiol Infect 139:1220-1229

246. Kemal J, Sibhat B, Abraham A, Terefe Y, Tulu KT, Welay K, Getahun N (2019) Bovine tuberculosis in eastern Ethiopia: prevalence, risk factors and its public health importance. BMC Infect Dis 19:9

247. Khattak I, Mushtaq MH, Ahmad MUD, Khan MS, Chaudhry M, Sadique U (2016) Risk factors associated with *Mycobacterium bovis* skin positivity in cattle and buffalo in Peshawar, Pakistan. Trop Anim Health Prod 48:479-485

248. King HC, Murphy A, James P, Travis E, Porter D, Hung YJ, Sawyer J, Cork J, Delahay RJ, Gaze W, Courtenay O, Wellington EM (2015) The variability and seasonality of the environmental reservoir of *Mycobacterium bovis* shed by wild European badgers. Sci Rep 5:7

249. Kjaer LJ, Schauber EM, Nielsen CK (2008) Spatial and temporal analysis of contact rates in female white-tailed deer. J Wildl Manag 72:1819-1825

250. Knight MA, Hutchings MR, White PCL, Davidson RS, Marion G (2022) A mechanistic model captures livestock trading, disease dynamics, and compensatory behaviour in response to control measures. J Theoret Biol 539:15

251. Knust BM, Wolf PC, Wells SJ (2011) Characterization of the risk of deer-cattle interactions in Minnesota by use of an on-farm environmental assessment tool. Am J Vet Res 72:924-931

252. Korniienko LY, Pyskun AV, Ukhovskyi VV, Karpulenko MS, Moroz OA, Pyskun OO, Tsarenko TM, Aliekseieva GB (2021) Retrospective analysis of the control and prevention of tuberculosis among cattle in Ukraine in the period 1994-2020. Regul Mech Biosyst 12:301-306

253. Kukielka E, Barasona JA, Cowie CE, Drewe JA, Gortazar C, Cotarelo I, Vicente J (2013) Spatial and temporal interactions between livestock and wildlife in South Central Spain assessed by camera traps. Prev Vet Med 112:213-221

254. LaHue NP, Banos JV, Acevedo P, Gortazar C, Martinez-Lopez B (2016) Spatially explicit modeling of animal tuberculosis at the wildlife-livestock interface in Ciudad Real province, Spain. Prev Vet Med 128:101-111

255. Langton TES, Jones MW, McGill I (2022) Analysis of the impact of badger culling on bovine tuberculosis in cattle in the high-risk area of England, 2009-2020. Vet Rec 190:13

256. Lavelle MJ, Campa H, LeDoux K, Ryan PJ, Fischer JW, Pepin KM, Blass CR, Glow MP, Hygnstrom SE, VerCauteren KC (2015) Deer response to exclusion from stored cattle feed in Michigan, USA. Prev Vet Med 121:159-164

257. Lavelle MJ, Kay SL, Pepin KM, Grear DA, Campa H, VerCauteren KC (2016) Evaluating wildlife-cattle contact rates to improve the understanding of dynamics of bovine tuberculosis transmission in Michigan, USA. Prev Vet Med 135:28-36

258. le Roex N, Cooper D, van Helden PD, Hoal EG, Jolles AE (2016) disease control in wildlife: evaluating a test and cull programme for bovine tuberculosis in african buffalo. Transbound Emerg Dis 63:647-657

259. Leghari A, Kamboh AA, Lakho SA, Khand FM, Malhi KK, Chandio IB, Baloch S, Shah JM (2020) Prevalence and risk factors associated with bovine tuberculosis in cattle in Hyderabad and Tando Allahyar Districts, Sindh, Pakistan. Pakistan J Zool 52:207-212

260. Lekko YM, Che-Amat A, Ooi PT, Omar S, Mohd-Hamdan DT, Linazah LS, Zakaria Z, Ramanoon SZ, Mazlan M, Jesse FFA, Abdul-Razak MFA, Jasni S, Abdul-Hamid N (2021) Detection of *Mycobacterium tuberculosis complex* antibodies in free-ranged wild boar and wild macaques in selected districts in Selangor and reevaluation of tuberculosis serodetection in captive Asian elephants in Pahang, Peninsular Malaysia. JVMS 83:1702-1707

261. Lintott RA, Norman RA, Hoyle AS (2013) The impact of increased dispersal in response to disease control in patchy environments. J Theor Biol 323:57-68

262. Liu YH, Kang Q, Yang B, Li F, Li XQ, Zhang L, Zhao L (2019) Prevalence of bovine tuberculosis in the Aksu Region of Xinjiang, China, between 1985 and 2016. Arq Bras Med Vet 71:374-378

263. Maciel ALG, Loiko MR, Bueno TS, Moreira JG, Coppola M, Dalla Costa ER, Schmid KB, Rodrigues RO, Cibulski SP, Bertagnolli AC, Mayer FQ (2018) Tuberculosis in Southern Brazilian wild boars (*Sus scrofa*): First epidemiological findings. Transbound Emerg Dis 65:518-526

264. Madden JM, McGrath G, Sweeney J, Murray G, Tratalos JA, More SJ (2021) Spatio-temporal models of bovine tuberculosis in the Irish cattle population, 2012-2019. Spat Spatiotemporal Epidemiol 39:11

265. Madeira S, Manteigas A, Ribeiro R, Otte J, Fonseca AP, Caetano P, Abernethy D, Boinas F (2017) Factors that influence *Mycobacterium bovis* Infection in red deer and wild boar in an epidemiological risk area for tuberculosis of game species in Portugal. Transbound Emerg Dis 64:793-804

266. Marangon S, Martini M, Dalla Pozza M, Neto JF (1998) A case-control study on bovine tuberculosis in the Veneto Region (Italy). Prev Vet Med 34:87-95

267. Mareledwane VE, Adesiyun AA, Thompson PN, Hlokwe TM (2021) Application of the gamma-interferon assay to determine the prevalence of bovine tuberculosis in slaughter livestock at abattoirs in Gauteng, South Africa. Vet Med Sci 8:2568-2575

268. Marsot M, Beral M, Scoizec A, Mathevon Y, Durand B, Courcoul A (2016) Herd-level risk factors for bovine tuberculosis in French cattle herds. Prev Vet Med 131:31-40

269. Martin SW, Eves JA, Dolan LA, Hammond RF, Griffin JM, Collins JD, Shoukri MM (1997) The association between the bovine tuberculosis status of herds in the East Offaly Project Area, and the distance to badger setts, 1988-1993. Prev Vet Med 31:113-125

270. Martin SW, O'Keeffe J, Byrne AW, Rosen LE, White PW, McGrath G (2020) Is moving from targeted culling to BCG-vaccination of badgers (*Meles meles*) associated with an unacceptable increased incidence of cattle herd tuberculosis in the Republic of Ireland? A practical non-inferiority wildlife intervention study in the Republic of Ireland (2011-2017). Prev Vet Med 179:15

271. Martinez-Guijosa J, Lima-Barbero JF, Acevedo P, Cano-Terriza D, Jimenez-Ruiz S, Barasona JA, Boadella M, Garcia-Bocanegra I, Gortazar C, Vicente J (2021) Description and implementation of an On-farm Wildlife Risk Mitigation Protocol at the wildlife-livestock interface: Tuberculosis in Mediterranean environments. Prev Vet Med 191:11

272. Martinez-Guijosa J, Lopez-Alonso A, Gortazar C, Acevedo P, Torres MJ, Vicente J (2021) Shared use of mineral supplement in extensive farming and its potential for infection transmission at the wildlife-livestock interface. Eur J Wildl Res 67:9

273. Martinez-Lopez B, Barasona JA, Gortazar C, Rodriguez-Prieto V, Sanchez-Vizcaino JM, Vicente J (2014) Farm-level risk factors for the occurrence, new infection or persistence of tuberculosis in cattle herds from South-Central Spain. Prev Vet Med 116:268-278

274. Mathews F, Lovett L, Rushton S, Macdonald DW (2006) Bovine tuberculosis in cattle: Reduced risk on wildlife-friendly farms. Biol Lett 2:271-274

275. Mazari MQ, Kalhoro DH, Baloch H, Kalhoro MS, Abro SH, Buriro R, Kaka A, Parveen F, Mangi MH, Lochi GM, Soomro AA, Soomro AG, Abbasi AG, Depar SH (2022) Prevalence and risk factors of bovine tuberculosis in cattle and dairy farm workers in Mirpurkhas and Badin Districts of Sindh, Pakistan. Pakistan J Zool 54:1115-1122

276. McCarty CW, Miller MW (1998) A versatile model of disease transmission applied to forecasting bovine tuberculosis dynamics in white-tailed deer populations. J Wildl Dis 34:722-730

277. McCluskey B, Lombard J, Strunk S, Nelson D, Robbe-Austerman S, Naugle A, Edmondson A (2014) *Mycobacterium bovis* in California dairies: A case series of 2002-2013 outbreaks. Prev Vet Med 115:205-216

278. McGrath G, Abernethy D, Stringer L, More SJ (2009) An all-island approach to mapping bovine tuberculosis in Ireland. Irish Vet J 62:192-197

279. McGrath G, Clegg TA, More SJ (2014) Recent spatial changes in bovine tuberculosis in the Republic of Ireland. Vet Rec 175:45-46

280. McInerney J, Small KJ, Caley P (1995) Prevalence of *Mycobacterium bovis* infection in feral pigs in the Northern Territory. Australian Vet J 72:448-451

281. McKenzie JS, Morris RS, Pfeiffer DU, Dymond JR (2002) Application of remote sensing to enhance the control of wildlife-associated *Mycobacterium bovis* infection. PE&RS 68:153-159

282. McKinley TJ, Lipschutz-Powell D, Mitchell AP, Wood JLN, Conlan AJK (2018) Risk factors and variations in detection of new bovine tuberculosis breakdowns via slaughterhouse surveillance in Great Britain. PLoS One 13:14

283. Mekonnen GA, Ameni G, Wood JLN, The ETHICOBOTS consortium, Berg S, Conlan AJK (2019) Network analysis of dairy cattle movement and associations with bovine tuberculosis spread and control in emerging dairy belts of Ethiopia. BMC Vet Res 15:14

284. Mekonnen GA, Conlan AJK, Berg S, Ayele BT, Alemu A, Guta S, Lakew M, Tadesse B, Gebre S, Wood JLN, Ameni G, The ETHICOBOTS consortium (2019) Prevalence of bovine tuberculosis and its associated risk factors in the emerging dairy belts of regional cities in Ethiopia. Prev Vet Med 168:81-89

285. Mekonnen GA, Conlan AJK, Berg S, Ayele BT, Mihret A, Olani A, Asgedom H, Aseffa A, Wood JLN, Ameni G (2021) Dynamics and risk of transmission of bovine tuberculosis in the emerging dairy regions of Ethiopia. Epidemiol Infect 149:9

286. Mentaberre G, Romero B, de Juan L, Navarro-Gonzalez N, Velarde R, Mateos A, Marco I, Olive-Boix X, Dominguez L, Lavin S, Serrano E (2014) Long-term assessment of wild boar harvesting and cattle removal for bovine tuberculosis control in free ranging populations. PLoS One 9:12

287. Menzies FD, Abernethy DA, Stringer LA, Honhold N, Gordon AW (2012) A matched cohort study investigating the risk of *Mycobacterium bovis* infection in the progeny of infected cows. Vet J 194:299-302

288. Menzies FD, Abernethy DA, Stringer LA, Jordan C (2011) A comparison of badger activity in two areas of high and low bovine tuberculosis incidence of Northern Ireland. Vet Microbiol 151:112-119

289. Menzies FD, McCormick CM, O'Hagan MJH, Collins SF, McEwan J, McGeown CF, McHugh GE, Hart CD, Stringer LA, Molloy C, Burns G, McBride SJ, Doyle LP, Courcier EA, McBride KR, McNair J, Thompson S, Corbett DM, Harwood RG, Trimble NA (2021) Test and vaccinate or remove: Methodology and preliminary results from a badger intervention research project. Vet Rec 189:12

290. Meunier NV, Sebulime P, White RG, Kock R (2017) Wildlife-livestock interactions and risk areas for cross-species spread of bovine tuberculosis. Onderstepoort J Vet Res 84:10

291. Milian-Suazo F, Salman MD, Ramirez C, Payeur JB, Rhyan JC, Santillan M (2000) Identification of tuberculosis in cattle slaughtered in Mexico. American J Vet Res 61:86-89

292. Mill AC, Rushton SP, Shirley MDF, Murray AWA, Smith GC, Delahay RJ, McDonald RA (2012) Farm-scale risk factors for bovine tuberculosis incidence in cattle herds during the Randomized Badger Culling Trial. Epidemiol Infect 140:219-230

293. Miller R, Kaneene JB, Fitzgerald SD, Schmitt SM (2003) Evaluation of the influence of supplemental feeding of white-tailed deer (*Odocoileus virginianus*) on the prevalence of bovine tuberculosis in the Michigan wild deer population. J Wildl Dis 39:84-95

294. Miller R, Kaneene JB, Schmitt SM, Lusch DP, Fitzgerald SD (2007) Spatial analysis of Mycobacterium bovis infection in white-tailed deer (*Odocoileus virginianus*) in Michigan, USA. Prev Vet Med 82:111-122

295. Miller RA, Kaneene JB (2006) Evaluation of historical factors influencing the occurrence and distribution of *Mycobacterium bovis* infection among wildlife in Michigan. Am J Vet Res 67:604-615

296. Milne G, Allen A, Graham J, Kirke R, McCormick C, Presho E, Skuce R, Byrne AW (2020) *Mycobacterium bovis* population structure in cattle and local badgers: co-localisation and variation by farm type. Pathogens 9:18

297. Milne G, Allen A, Graham J, Lahuerta-Marin A, McCormick C, Presho E, Reid N, Skuce R, Byrne AW (2020) Bovine tuberculosis breakdown duration in cattle herds: an investigation of herd, host, pathogen and wildlife risk factors. Peerj 8:24

298. Milne GM, Graham J, Allen A, Lahuerta-Marin A, McCormick C, Presho E, Skuce R, Byrne AW (2019) Spatiotemporal analysis of prolonged and recurrent bovine tuberculosis breakdowns in Northern Irish cattle herds reveals a new infection hotspot. Spat Spatiotemporal Epidemiol 28:33-42

299. Milne MG, Graham J, Allen A, McCormick C, Presho E, Skuce R, Byrne AW (2019) Variation in *Mycobacterium bovis* genetic richness suggests that inwards cattle movements are a more important source of infection in beef herds than in dairy herds. BMC Microbiol 19:13

300. Moiane I, Machado A, Santos N, Nhambir A, Inlamea O, Hattendorf J, Kallenius G, Zinsstag J, Correia-Neves M (2014) Prevalence of bovine tuberculosis and risk factor assessment in cattle in rural livestock areas of Govuro District in the Southeast of Mozambique. PLoS One 9:9

301. More SJ, Houtsma E, Doyle L, McGrath G, Clegg TA, de la Rua-Domenech R, Duignan A, Blissitt MJ, Dunlop M, Schroeder PG, Pike R, Upton P (2018) Further description of bovine tuberculosis trends in the United Kingdom and the Republic of Ireland, 2003-2015. Vet Rec 183:22

302. Moustakas A, Evans MR (2015) Coupling models of cattle and farms with models of badgers for predicting the dynamics of bovine tuberculosis (TB). SERRA 29:623-635

303. Moustakas A, Evans MR (2016) Regional and temporal characteristics of bovine tuberculosis of cattle in Great Britain. SERRA 30:989-1003

304. Moustakas A, Evans MR (2017) A big-data spatial, temporal and network analysis of bovine tuberculosis between wildlife (badgers) and cattle. SERRA 31:315-328

305. Moustakas A, Evans MR, Daliakopoulos IN, Markonis Y (2018) Abrupt events and population synchrony in the dynamics of Bovine Tuberculosis. Nature Commun 9:10

306. Mukherjee F (2006) Comparative prevalence of tuberculosis in two dairy herds in India. OMSA 25:1125-1130

307. Mullen EM, MacWhite T, Maher PK, Kelly DJ, Marples NM, Good M (2013) Foraging Eurasian badgers *Meles meles* and the presence of cattle in pastures. Do badgers avoid cattle? Appl Anim Behav Sci 144:130-137

308. Mullen EM, MacWhite T, Maher PK, Kelly DJ, Marples NM, Good M (2015) The avoidance of farmyards by European badgers *Metes meles* in a medium density population. Appl Anim Behav Sci 171:170-176

309. Munroe FA, Dohoo IR, McNab WB (2000) Estimates of within-herd incidence rates of *Mycobacterium bovis* in Canadian cattle and cervids between 1985 and 1994. Prev Vet Med 45:247-256

310. Munroe FA, Dohoo IR, McNab WB, Spangler L (1999) Risk factors for the between-herd spread of *Mycobacterium bovis* in Canadian cattle and cervids between 1985 and 1994. Prev Vet Med 41:119-133

311. Munyeme M, Muma JB, Samui KL, Skjerve E, Nambota AM, Phiri IGK, Rigouts L, Tryland M (2009) Prevalence of bovine tuberculosis and animal level risk factors for indigenous cattle under different grazing strategies in the livestock/wildlife interface areas of Zambia. Trop Anim Health Prod 41:345-352

312. Munyeme M, Muma JB, Skjerve E, Nambota AM, Phiri IGK, Samui KL, Dorny P, Tryland M (2008) Risk factors associated with bovine tuberculosis in traditional cattle of the livestock/wildlife interface areas in the Kafue basin of Zambia. Prev Vet Med 85:317-328

313. Murphy KJ, Morera-Pujol V, Ryan E, Byrne AW, Breslin P, Ciuti S (2022) Habitat availability alters the relative risk of a bovine tuberculosis breakdown in the aftermath of a commercial forest clearfell disturbance. J Appl Ecol 59:2333–2345

314. Mwakapuja RS, Makondo ZE, Malakalinga J, Bryssinckx W, Mdegela RH, Moser I, Kazwala RR, Tanner M (2013) Prevalence and significant geospatial clusters of bovine tuberculosis infection at livestock-wildlife interface ecosystem in Eastern Tanzania. Trop Anim Health Prod 45:1223-1230

315. Nalapa DP, Muwonge A, Kankya C, Olea-Popelka F (2017) Prevalence of tuberculous lesion in cattle slaughtered in Mubende district, Uganda. BMC Vet Res 13:8

316. Nespoli JMB, Negreiros RL, Amaku M, Dias RA, Ferreira F, Telles EO, Heinemann MB, Grisi JHH, Goncalves VSP, Neto JSF (2016) Epidemiological situation of bovine tuberculosis in the state of Mato Grosso, Brazil. Semin Cienc Agrar 37:3589-3599

317. Nikolovski G, Petrov EA, Cokrevski S, Arsevska E, Nikolovska G (2012) Bovine tuberculosis in cattle during the implementation of official control measures in Republic of Macedonia for the period 2007-2009. Slovenian Vet Res 49:79-87

318. Nol P, Rhyan JC, Robbe-Austerman S, McCollum MP, Rigg TD, Saklou NT, Salman MD (2013) The potential for transmission of BCG from orally vaccinated white-tailed deer (*Odocoileus virginianus*) to cattle (*Bos taurus*) through a contaminated environment: experimental findings. PLoS One 8:5

319. Norton S, Corner LAL, Morris RS (2005) Ranging behaviour and duration of survival of wild brushtail possums (*Trichosurus vulpecula*) infected with *Mycobacterium bovis*. New Zealand Vet J 53:293-300

320. Nugent G, Whitford J, Yockney IJ, Cross ML (2012) Reduced spillover transmission of *Mycobacterium bovis* to feral pigs (*Sus scofa*) following population control of brushtail possums (*Trichosurus vulpecula*). Epidemiol Infect 140:1036-1047

321. Nugent G, Yockney IJ, Whitford EJ, Cross ML, Aldwell FE, Buddle BM (2016) Field trial of an aerially-distributed tuberculosis vaccine in a low-density wildlife population of brushtail possums (*Trichosurus vulpecula*). PLoS One 11:19

322. O Mairtin D, Williams DH, Dolan L, Eves JA, Collins JD (1998) The influence of selected herd factors and a badger-intervention tuberculosis-control programme on the risk of a herd-level trade restriction to a bovine population in Ireland. Prev Vet Med 35:79-90

323. O Mairtin D, Williams DH, Griffin JM, Dolan LA, Eves JA (1998) The effect of a badger removal programme on the incidence of tuberculosis in an Irish cattle population. Prev Vet Med 34:47-56

324. O'Brien DJ, Schmitt SM, Fierke JS, Hogle SA, Winterstein SR, Cooley TM, Moritz WE, Diegel KL, Fitzgerald SD, Berry DE, Kaneene JB (2002) Epidemiology of *Mycobacterium bovis* in free-ranging white-tailed deer, Michigan, USA, 1995-2000. Prev Vet Med 54:47-63

325. O'Hagan MJH, Courcier EA, Drewe JA, Gordon AW, McNair J, Abernethy DA (2015) Risk factors for visible lesions or positive laboratory tests in bovine tuberculosis reactor cattle in Northern Ireland. Prev Vet Med 120:283-290

326. O'Hagan MJH, Gordon AW, McCormick CM, Collins SF, Trimble NA, McGeown CF, McHugh GE, McBride KR, Menzies FD (2021) Effect of selective removal of badgers (*Meles meles*) on ranging behaviour during a 'Test and Vaccinate or Remove' intervention in Northern Ireland. Epidemiol Infect 149:9

327. O'Hagan MJH, Matthews DI, Laird C, McDowell SWJ (2016) Herd-level risk factors for bovine tuberculosis and adoption of related biosecurity measures in Northern Ireland: A case-control study. Vet J 213:26-32

328. O'Hagan MJH, McCormick CM, Collins SF, McBride KR, Menzies FD (2021) Are major roads effective barriers for badger (*Meles meles*) movements? Res J Vet Sci 138:49-52

329. O'Hagan MJH, Stegeman JA, Doyle LP, Stringer LA, Courcier EA, Menzies FD (2018) The impact of the number of tuberculin skin test reactors and infection confirmation on the risk of future bovine tuberculosis incidents; a Northern Ireland perspective. Epidemiol Infect 146:1495-1502

330. O'Hare A, Balaz D, Wright DM, McCormick C, McDowell S, Trewby H, Skuce RA, Kao RR (2021) A new phylodynamic model of *Mycobacterium bovis* transmission in a multi-host system uncovers the role of the unobserved reservoir. PLoS Comput Biol 17:15

331. O'Hare A, Orton RJ, Bessell PR, Kao RR (2014) Estimating epidemiological parameters for bovine tuberculosis in British cattle using a Bayesian partial-likelihood approach. Proc Biol Sci 281:9

332. O'Mahony DT (2014) Use of water troughs by badgers and cattle. Vet J 202:628-629

333. O'Mahony DT (2015) Badger (*Meles meles*) contact metrics in a medium-density population. Mammal Biol 80:484-490

334. O'Mahony DT (2015) Multi-species visit rates to farmyards: Implications for biosecurity. Vet J 203:126-128

335. OcorryCrowe G, Hammond R, Eves J, Hayden TJ (1996) The effect of reduction in badger density on the spatial organisation and activity of badgers *Meles meles* *L.* in relation to farms in central Ireland. B&E 96B:147-158

336. Olea-Popelka F, Butler D, Lavin D, McGrath G, O'Keeffel J, Kelton D, Berke O, More S, Martin W (2006) A case study of bovine tuberculosis in an area of County Donegal, Ireland. Irish Vet J 59:683-690

337. Olea-Popelka FJ, Costello E, White P, McGrath G, Collins JD, O'Keeffe J, Kelton DF, Berke O, More S, Martin SW (2008) Risk factors for disclosure of additional tuberculous cattle in attested-clear herds that had one animal with a confirmed lesion of tuberculosis at slaughter during 2003 in Ireland. Prev Vet Med 85:81-91

338. Olea-Popelka FJ, Fitzgerald P, White P, McGrath G, Collins JD, O'Keeffe J, Kelton DF, Berke O, More S, Martin SW (2009) Targeted badger removal and the subsequent risk of bovine tuberculosis in cattle herds in county Laois, Ireland. Prev Vet Med 88:178-184

339. Olea-Popelka FJ, Flynn O, Costello E, McGrath G, Collins JD, O'Keeffe J, Kelton DF, Berke O, Martin SW (2005) Spatial relationship between *Mycobacterium bovis* strains in cattle and badgers in four areas in Ireland. Prev Vet Med 71:57-70

340. Olea-Popelka FJ, Griffin JM, Collins JD, McGrath G, Martin SW (2003) Bovine tuberculosis in badgers in four areas in Ireland: does tuberculosis cluster? Prev Vet Med 59:103-111

341. Olea-Popelka FJ, Phelan J, White PW, McGrath G, Collins JD, O'Keeffe J, Duggan M, Collins DM, Kelton DF, Berke O, More SJ, Martin SW (2006) Quantifying badger exposure and the risk of bovine tuberculosis for cattle herds in county Kilkenny, Ireland. Prev Vet Med 75:34-46

342. Olea-Popelka FJ, White PW, Collins JD, O'Keeffe J, Kelton DE, Martin SW (2004) Breakdown severity during a bovine tuberculosis episode as a predictor of future herd breakdowns in Ireland. Prev Vet Med 63:163-172

343. Oloya J, Muma JB, Opuda-Asibo J, Djonne B, Kazwala R, Skjerve E (2007) Risk factors for herd-level bovine-tuberculosis seropositivity in transhumant cattle in Uganda. Prev Vet Med 80:318-329

344. Oloya J, Opuda-Asibo J, Djonne B, Muma JB, Matope G, Kazwala R, Skjerve E (2006) Responses to tuberculin among Zebu cattle in the transhumance regions of Karamoja and Nakasongola district of Uganda. Trop Anim Health Prod 38:275-283

345. Omer MK, Skjerve E, Woldehiwet Z, Holstad G (2001) A cross-sectional study of bovine tuberculosis in dairy farms in Asmara, Eritrea. Trop Anim Health Prod 33:295-303

346. Orrico M, van Schaik G, Koets A, van den Broek J, Montizaan M, La Haye M, Rijks JM (2022) The effectiveness of bovine tuberculosis surveillance in Dutch badgers. Transbound Emerg Dis 69:2008-2020

347. Orton RJ, Deason M, Bessell PR, Green DM, Kao RR, Salvador LCM (2018) Identifying genotype specific elevated-risk areas and associated herd risk factors for bovine tuberculosis spread in British cattle. Epidemics 24:34-42

348. Palisson A, Courcoul A, Durand B (2016) Role of Cattle Movements in Bovine Tuberculosis Spread in France between 2005 and 2014. PLoS One 11:19

349. Palmer MV, Waters WR, Whipple DL (2002) Milk containing *Mycobacterium bovis* as a source of infection for white-tailed deer fawns (*Odocoileus virginianus*). Tuberculosis 82:161-165

350. Palmer MV, Waters WR, Whipple DL (2004) Investigation of the transmission of *Mycobacterium bovis* from deer to cattle through indirect contact. Am J Vet Res 65:1483-1489

351. Palmer MV, Waters WR, Whipple DL (2004) Shared feed as a means of deer-to-deer transmission of *Mycobacterium bovis*. J Wildl Dis 40:87-91

352. Palmer MV, Whipple DL, Waters WR (2001) Experimental deer-to-deer transmission of *Mycobacterium bovis*. Am J Vet Res 62:692-696

353. Parra A, Garcia A, Inglis NF, Tato A, Alonso JM, de Mendoza MH, de Mendoza JH, Larrasa J (2006) An epidemiological evaluation of *Mycobacterium bovis* infections in wild game animals of the Spanish Mediterranean ecosystem. Res J Vet Sci 80:140-146

354. Pascual-Linaza AV, Gordon AW, Stringer LA, Menzies FD (2017) Efficiency of slaughterhouse surveillance for the detection of bovine tuberculosis in cattle in Northern Ireland. Epidemiol Infect 145:995-1005

355. Paterson BM, Morris RS (1995) Interactions between beef cattle and simulated tuberculous possums on pasture. New Zealand Vet J 43:289-293

356. Paterson BM, Morris RS, Weston J, Cowan PE (1995) Foraging and denning patterns of brushtail possums, and their possible relationship to contact with cattle and the transmission of bovine tuberculosis. New Zealand Vet J 43:281-288

357. Pavlik I, Ayele WY, Parmova I, Melicharek I, Hanzlikova M, Kormendy B, Nagy G, Cvetnic Z, Ocepek M, Fejzic N, Lipiec M (2002) Incidence of bovine tuberculosis in cattle in seven Central European countries during the years 1990-1999. Vet Med 47:45-51

358. Pavlik I, Trcka I, Parmova I, Svobodova J, Melicharek I, Nagy G, Cvetnic Z, Ocepek M, Pate M, Lipiec M (2005) Detection of bovine and human tuberculosis in cattle and other animals in six Central European countries during the years 2000-2004. Vet Med 50:291-299

359. Payne A, Boschiroli ML, Gueneau E, Moyen JL, Rambaud T, Dufour B, Gilot-Fromont E, Hars J (2013) Bovine tuberculosis in "Eurasian" badgers (*Meles meles*) in France. Eur J Wildl Res 59:331-339

360. Payne A, Chappa S, Hars J, Dufour B, Gilot-Fromont E (2016) Wildlife visits to farm facilities assessed by camera traps in a bovine tuberculosis-infected area in France. Eur J Wildl Res 62:33-42

361. Payne A, Philipon S, Hars J, Dufour B, Gilot-Fromont E (2017) Wildlife interactions on Baited Places and Waterholes in a French area infected by Bovine Tuberculosis. Front Vet Sci 3:11

362. Perea C, Ciaravino G, Stuber T, Thacker TC, Robbe-Austerman S, Allepuz A, de Val BP (2021) Whole-Genome SNP analysis identifies putative *Mycobacterium bovis* transmission clusters in livestock and wildlife in Catalonia, Spain. Microorganisms 9:24

363. Perez AM, Ward MP, Charmandarian A, Ritacco V (2002) Simulation model of within-herd transmission of bovine tuberculosis in Argentine dairy herds. Prev Vet Med 54:361-372

364. Perez AM, Ward MP, Torres P, Ritacco V (2002) Use of spatial statistics and monitoring data to identify clustering of bovine tuberculosis in Argentina. Prev Vet Med 56:63-74

365. Pfeiffer DU, Hickling GJ, Morris RS, Patterson KP, Ryan TJ, Crews KB (1995) The epidemiology of Mycobacterium bovis infection in brushtail possums (*Trichosurus vulpecula* Kerr) in the Hauhungaroa ranges, New Zealand. New Zealand Vet J 43:272-280

366. Phepa PB, Chirove F, Govinder KS (2016) Modelling the role of multi-transmission routes in the epidemiology of bovine tuberculosis in cattle and buffalo populations. Math Biosci 277:47-58

367. Phillips GE, Lavelle MJ, Fischer JW, White JJ, Wells SJ, VerCauteren KC (2012) A novel bipolar electric fence for excluding white-tailed deer from stored livestock feed. J Anim Sci 90:4090-4097

368. Picasso C, Alvarez J, VanderWaal KL, Fernandez F, Gil A, Wells SJ, Perez A (2017) Epidemiological investigation of bovine tuberculosis outbreaks in Uruguay (2011-2013). Prev Vet Med 138:156-161

369. Pope LC, Butlin RK, Wilson GJ, Woodroffe R, Erven K, Conyers CM, Franklin T, Delahay RJ, Cheeseman CL, Burke T (2007) Genetic evidence that culling increases badger movement: implications for the spread of bovine tuberculosis. Mol Ecol 16:4919-4929

370. Porphyre T, McKenzie J, Stevenson M (2007) A descriptive spatial analysis of bovine tuberculosis in intensively controlled cattle farms in New Zealand. BMC Vet Res 38:465-479

371. Porphyre T, McKenzie J, Stevenson MA (2011) Contact patterns as a risk factor for bovine tuberculosis infection in a free-living adult brushtail possum *Trichosurus vulpecula* population. Prev Vet Med 100:221-230

372. Porphyre T, Stevenson M, Jackson R, McKenzie J (2008) Influence of contact heterogeneity on TB reproduction ratio R-0 in a free-living brushtail possum Trichosurus vulpecula population. BMC Vet Res 39:13

373. Porphyre T, Stevenson MA, McKenzie J (2008) Risk factors for bovine tuberculosis in New Zealand cattle farms and their relationship with possum control strategies. Prev Vet Med 86:93-106

374. Portacci K, Lombard J, Schoenbaum M, Orloski K, Camacho M (2014) The occurrence of M. bovis cases in US cattle, 2001-2011. In: Thoen CO, Steele JH, Kaneene JB (eds) Zoonotic tuberculosis: *Mycobacterium bovis* and other pathogenic mycobacteria, Volume 3. Wiley, UK

375. Pozo P, Cardenas NC, Bezos J, Romero B, Grau A, Nacar J, Saez JL, Minguez O, Alvarez J (2021) Evaluation of the performance of slaughterhouse surveillance for bovine tuberculosis detection in Castilla y Leon, Spain. Prev Vet Med 189:14

376. Pozo P, Romero B, Bezos J, Grau A, Nacar J, Saez JL, Minguez O, Alvarez J (2020) Evaluation of Risk Factors Associated With Herds With an Increased Duration of Bovine Tuberculosis Breakdowns in Castilla y Leon, Spain (2010-2017). Front Vet Sci 7:14

377. Pozo P, VanderWaal K, Grau A, de la Cruz ML, Nacar J, Bezos J, Perez A, Minguez O, Alvarez J (2019) Analysis of the cattle movement network and its association with the risk of bovine tuberculosis at the farm level in Castilla y Leon, Spain. Transbound Emerg Dis 66:327-340

378. Prentice JC, Fox NJ, Hutchings MR, White PCL, Davidson RS, Marion G (2019) When to kill a cull: factors affecting the success of culling wildlife for disease control. J R Soc Interface 16:11

379. Price-Carter M, Brauning R, de Lisle GW, Livingstone P, Neill M, Sinclair J, Paterson B, Atkinson G, Knowles G, Crews K, Crispell J, Kao R, Robbe-Austerman S, Stuber T, Parkhill J, Wood J, Harris S, Collins DM (2018) Whole genome sequencing for determining the source of *Mycobacterium bovis* infections in livestock herds and wildlife in New Zealand. Front Vet Sci 5:13

380. Proano-Perez F, Benitez-Ortiz W, Celi-Erazo M, Ron-Garrido L, Benitez-Capistros R, Portaels F, Rigouts L, Linden A (2009) Comparative intradermal tuberculin test in dairy cattle in the North of Ecuador and risk factors associated with bovine tuberculosis. ASTMH 81:1103-1109

381. Queiros J, Vicente J, Alves PC, de la Fuente J, Gortazar C (2016) Tuberculosis, genetic diversity and fitness in the red deer, *Cervus elaphus*. Infect Genet Evol 43:203-212

382. Queiros J, Vicente J, Boadella M, Gortazar C, Alves PC (2014) The impact of management practices and past demographic history on the genetic diversity of red deer (*Cervus elaphus*): an assessment of population and individual fitness. Biol J Linn Soc 111:209-223

383. Queiroz MR, Groff ACM, Silva ND, Grisi JHH, Amaku M, Dias RA, Telles EO, Heinemann MB, Neto JSF, Goncalves VSP, Ferreira F (2016) Epidemiological status of bovine tuberculosis in the state of Rio Grande do Sul, Brazil. Semin Cienc Agrar 37:3647-3657

384. Ragg JR, Mackintosh CG, Moller H (2000) The scavenging behaviour of ferrets (*Mustela furo*), feral cats (*Felis domesticus*), possums (*Trichosurus vulpecula*), hedgehogs (*Erinaceus europaeus*) and harrier hawks (*Circus approximans*) on pastoral farmland in New Zealand: Implications for bovine tuberculosis transmission. New Zealand Vet J 48:166-175

385. Ramanujam H, Thiruvengadam K, Singaraj R, Palaniyandi K (2022) Role of abattoir monitoring in determining the prevalence of bovine tuberculosis: A systematic review and meta-analysis. Transbound Emerg Dis 69:958-973

386. Ramirez-Villaescusa AM, Medley GF, Mason S, Green LE (2010) Risk factors for herd breakdown with bovine tuberculosis in 148 cattle herds in the south west of England. Prev Vet Med 95:224-230

387. Ramos B, Pereira AC, Reis AC, Cunha MV (2020) Estimates of the global and continental burden of animal tuberculosis in key livestock species worldwide: A meta-analysis study. One Health 10:10

388. Ramsey D, Cowan P (2003) Mortality rate and movements of brushtail possums with clinical tuberculosis (*Mycobacterium bovis* infection). New Zealand Vet J 51:179-185

389. Ramsey D, Spencer N, Caley P, Efford M, Hansen K, Lam M, Cooper D (2002) The effects of reducing population density on contact rates between brushtail possums: implications for transmission of bovine tuberculosis. J Appl Ecol 39:806-818

390. Ramsey DSL, Coleman JD, Coleman MC, Horton P (2006) The effect of fertility control on the transmission of bovine tuberculosis in wild brushtail possums. New Zealand Vet J 54:218-223

391. Ramsey DSL, Efford MG (2010) Management of bovine tuberculosis in brushtail possums in New Zealand: predictions from a spatially explicit, individual-based model. J Appl Ecol 47:911-919

392. Ramsey DSL, O'Brien DJ, Cosgrove MK, Rudolph BA, Locher AB, Schmitt SM (2014) Forecasting eradication of bovine tuberculosis in Michigan white-tailed deer. J Wildl Manag 78:240-254

393. Ramsey DSL, O'Brien DJ, Smith RW, Cosgrove MK, Schmitt SM, Rudolph BA (2016) Management of on-farm risk to livestock from bovine tuberculosis in Michigan, USA, white-tailed deer: Predictions from a spatially-explicit stochastic model. Prev Vet Med 134:26-38

394. Regassa A, Tassew A, Amenu K, Megersa B, Abunna F, Mekibib B, Marcotty T, Ameni G (2010) A cross-sectional study on bovine tuberculosis in Hawassa town and its surroundings, Southern Ethiopia. Trop Anim Health Prod 42:915-920

395. Reilly LA, Courtenay O (2007) Husbandry practices, badger sett density and habitat composition as risk factors for transient and persistent bovine tuberculosis on UK cattle farms. Prev Vet Med 80:129-142

396. Reveillaud E, Desvaux S, Boschiroli ML, Hars J, Faure E, Fediaevsky A, Cavalerie L, Chevalier F, Jabert P, Poliak S, Tourette I, Hendrikx P, Richomme C (2018) Infection of wildlife by *Mycobacterium bovis* in France assessment through a nationa surveillance system, Sylvatub. Front Vet Sci 5:16

397. Ribeiro-Lima J, Carstensen M, Cornicelli L, Forester JD, Wells SJ (2017) Patterns of cattle farm visitation by white-tailed deer in relation to risk of disease transmission in a previously infected area with bovine tuberculosis in Minnesota, USA. Transbound Emerg Dis 64:1519-1529

398. Ribeiro-Lima J, Enns EA, Thompson B, Craft ME, Wells SJ (2015) From network analysis to risk analysis-An approach to risk-based surveillance for bovine tuberculosis in Minnesota, US. Prev Vet Med 118:328-340

399. Ribeiro-Lima J, Schwabenlander S, Oakes M, Thompson B, Wells SJ (2016) Risk profiling of cattle farms as a potential tool in risk-based surveillance for *Mycobacterium bovis* infection among cattle in tuberculosis-free areas. JAVMA 248:1404-1413

400. Riordan P, Delahay RJ, Cheeseman C, Johnson PJ, Macdonald DW (2011) Culling-induced changes in badger (*Meles meles*) behaviour, social organisation and the epidemiology of bovine tuberculosis. PLoS One 6:9

401. Roberts MG (1992) The dynamics and control of bovine tuberculosis in possums. Ima J Math Med Biol 9:19-28

402. Roberts MG (1996) The dynamics of bovine tuberculosis in possum populations, and its eradication or control by culling or vaccination. J Anim Ecol 65:451-464

403. Roberts MG, Saha AK (1999) The asymptotic behaviour of a logistic epidemic model with stochastic disease transmission. Appl Math Lett 12:37-41

404. Robertson A, Judge J, Wilson GJ, Vernon IJ, Delahay RJ, McDonald RA (2019) Predicting badger visits to farm yards and making predictions available to farmers. PLoS One 14:20

405. Rocha WV, Jayme VD, Mota A, de Brito W, Pires GRD, Neto JSF, Grisi JHH, Dias RA, Amaku M, Telles EO, Ferreira F, Heinemann MB, Picão Gonçalves VS (2016) Prevalence and herd-level risk factors of bovine tuberculosis in the State of Goias, Brazil. Semin Cienc Agrar 37:3625-3638

406. Rodriguez-Prieto V, Martinez-Lopez B, Barasona JA, Acevedo P, Romero B, Rodriguez-Campos S, Gortazar C, Sanchez-Vizcaino JM, Vicente J (2012) A Bayesian approach to study the risk variables for tuberculosis occurrence in domestic and wild ungulates in South Central Spain. BMC Vet Res 8:13

407. Rodwell TC, Kriek NP, Bengis RG, Whyte IJ, Viljoen PC, de Vos V, Boyce WM (2001) Prevalence of bovine tuberculosis in African buffalo at Kruger National Park. J Wildl Dis 37:258-264

408. Rodwell TC, Whyte IJ, Boyce WM (2001) Evaluation of population effects of bovine tuberculosis in free-ranging African buffalo (*Syncerus caffer*). J Mammal 82:231-238

409. Rogers LM, Delahay R, Cheeseman CL, Langton S, Smith GC, Clifton-Hadley RS (1998) Movement of badgers (*Meles meles*) in a high-density population: individual, population and disease effects. Proc Biol Sci 265:1269-1276

410. Rogers LM, Forrester GJ, Wilson GJ, Yarnell RW, Cheeseman CL (2003) The role of setts in badger (*Meles meles*) group size, breeding success and status of TB (*Mycobacterium bovis*). J Zool 260:209-215

411. Romero MP, Chang YM, Brunton LA, Parry J, Prosser A, Upton P, Rees E, Tearne O, Arnold M, Stevens K, Drewe JA (2020) Decision tree machine learning applied to bovine tuberculosis risk factors to aid disease control decision making. Prev Vet Med 175:12

412. Rossi G, Aubry P, Dube C, Smith RL (2019) The spread of bovine tuberculosis in Canadian shared pastures: Data, model, and simulations. Transbound Emerg Dis 66:562-577

413. Rossi G, Crispell J, Balaz D, Lycett SJ, Benton CH, Delahay RJ, Kao RR (2020) Identifying likely transmissions in *Mycobacterium bovis* infected populations of cattle and badgers using the Kolmogorov Forward Equations. Sci Rep 10:13

414. Rouco C, Jewell C, Richardson KS, French NP, Buddle BM, Tompkins DM (2018) Brushtail possum (*Trichosurus vulpecula*) social interactions and their implications for bovine tuberculosis epidemiology. Behaviour 155:621-637

415. Rouco C, Norbury GL, Anderson DP (2017) Movements and habitat preferences of pests help to improve population control: the case of common brushtail possums in a New Zealand dryland ecosystem. Pest Manag Sci 73:287-294

416. Rouco C, Richardson KS, Buddle BM, French NP, Tompkins DM (2016) Sex difference in the survival rate of wild brushtail possums (*Trichosurus vulpecula*) experimentally challenged with bovine tuberculosis. Res J Vet Sci 107:102-105

417. Roug A, Clifford D, Mazet J, Kazwala R, John J, Coppolillo P, Smith W (2014) Spatial predictors of bovine tuberculosis infection and *Brucella* spp. exposure in pastoralist and agropastoralist livestock herds in the Ruaha ecosystem of Tanzania. Trop Anim Health Prod 46:837-843

418. Ruget AS, Rossi G, Pepler PT, Beaunee G, Banks CJ, Enright J, Kao RR (2021) Multi-species temporal network of livestock movements for disease spread. Appl Netw Sci 6:20

419. Sa'idu AS, Mohammed S, Ashafa M, Gashua MM, Mahre MB, Maigado AI (2017) Retrospective study of bovine tuberculosis in Gombe Township Abattoir, Northeastern Nigeria. Int J Vet Sci Med 5:65-69

420. Salvador LCM, Deason M, Enright J, Bessell PR, Kao RR (2018) Risk-based strategies for surveillance of tuberculosis infection in cattle for low-risk areas in England and Scotland. Epidemiol Infect 146:107-118

421. Salvador LCM, O'Brien DJ, Cosgrove MK, Stuber TP, Schooley AM, Crispell J, Church SV, Grohn YT, Robbe-Austerman S, Kao RR (2019) Disease management at the wildlife-livestock interface: Using whole-genome sequencing to study the role of elk in *Mycobacterium bovis* transmission in Michigan, USA. Mol Ecol 28:2192-2205

422. Santos N, Correia-Neves M, Ghebremichael S, Kallenius G, Svenson SB, Almeida V (2009) Epidemiology of *Mycobacterium bovis* infection in wild boar (*Sus scrofa*) from Portugal. J Wildl Dis 45:1048-1061

423. Santos N, Nunes T, Fonseca C, Vieira-Pinto M, Almeida V, Gortazar C, Correia-Neves M (2018) Spatial analysis of wildlife tuberculosis based on a serologic survey using dried blood spots, Portugal. Emerg Infect Dis 24:2169-2175

424. Santos N, Richomme C, Nunes T, Vicente J, Alves PC, de la Fuente J, Correia-Neves M, Boschiroli ML, Delahay R, Gortazar C (2020) Quantification of the animal tuberculosis multi-host community offers insights for control. Pathogens 9:11

425. Sauter CM, Morris RS (1995) Dominance hierarchies in cattle and red deer (*Cervus elaphus*): Their possible relationship to the transmission of bovine tuberculosis. New Zealand Vet J 43:301-305

426. Scantlebury M, Harris S, Allcroft DJ, Hutchings MR (2006) Individual trade-offs between nutrition and risk of interspecific transmission of disease by grazing: cows, badger latrines and bovine tuberculosis. Behaviour 143:141-158

427. Scantlebury M, Hutchings MR, Allcroft DJ, Harris S (2004) Risk of disease from wildlife reservoirs: Badgers, cattle, and bovine tuberculosis. JDS 87:330-339

428. Schauber EM, Nielsen CK, Kjaer LJ, Anderson CW, Storm DJ (2015) Social affiliation and contact patterns among white-tailed deer in disparate landscapes: implications for disease transmission. J Mammal 96:16-28

429. Schauber EM, Storm DJ, Nielsen CK (2007) Effects of joint space use and group membership on contact rates among white-tailed deer. J Wildl Manag 71:155-163

430. Schoning JM, Cerny N, Prohaska S, Wittenbrink MM, Smith NH, Bloemberg G, Pewsner M, Schiller I, Origgi FC, Ryser-Degiorgis MP (2013) Surveillance of bovine tuberculosis and risk estimation of a future reservoir formation in wildlife in Switzerland and Liechtenstein. PLoS One 8:13

431. Schroeder P, Hopkins B, Jones J, Galloway T, Pike R, Rolfe S, Hewinson G (2020) Temporal and spatial *Mycobacterium bovis* prevalence patterns as evidenced in the All Wales Badgers Found Dead (AWBFD) survey of infection 2014-2016. Sci Rep 10:11

432. Sedighi T, Varga L (2021) Evaluating the bovine tuberculosis eradication mechanism and its risk factors in England's cattle farms. Int J Environ Res Public Health 18:24

433. Shirima GM, Kazwala RR, Kambarage DM (2003) Prevalence of bovine tuberculosis in cattle in different farming systems in the eastern zone of Tanzania. Prev Vet Med 57:167-172

434. Shirley MDF, Rushtown SP, Smith GC, South AB, Lurz PWW (2003) Investigating the spatial dynamics of bovine tuberculosis in badger populations: evaluating an individual-based simulation model. Ecol Modell 167:139-157

435. Shitaye JE, Getahun B, Alemayehu T, Skoric M, Treml F, Fictum P, Vrbas V, Pavlik I (2006) A prevalence study of bovine tuberculosis by using abattoir meat inspection and tuberculin skin testing data, histopathological and IS6110PCR examination of tissues with tuberculous lesions in cattle in Ethiopia. Vet Med 51:512-522

436. Sichewo PR, Etter EMC, Michel AL (2019) Prevalence of *Mycobacterium bovis* infection in traditionally managed cattle at the wildlife-livestock interface in South Africa in the absence of control measures. Vet Res Commun 43:155-164

437. Sichewo PR, Etter EMC, Michel AL (2020) Wildlife-cattle interactions emerge as drivers of bovine tuberculosis in traditionally farmed cattle. Prev Vet Med 174:8

438. Silk MJ, Drewe JA, Delahay RJ, Weber N, Steward LC, Wilson-Aggarwal J, Boots M, Hodgson DJ, Croft DP, McDonald RA (2018) Quantifying direct and indirect contacts for the potential transmission of infection between species using a multilayer contact network. Behaviour 155:731-757

439. Silk MJ, Weber N, Steward LC, Delahay RJ, Croft DP, Hodgson DJ, Boots M, McDonald RA (2017) Seasonal variation in daily patterns of social contacts in the European badger *Meles meles*. Nat Ecol Evol 7:9006-9015

440. Silk MJ, Weber NL, Steward LC, Hodgson DJ, Boots M, Croft DP, Delahay RJ, McDonald RA (2018) Contact networks structured by sex underpin sex-specific epidemiology of infection. Ecol Lett 21:309-318

441. Silva MDP, Goncalves VSP, Mota A, Koloda M, Neto JSF, Grisi JHH, Dias RA, Amaku M, Telles EO, Ferreira F, Heinemann MB, Alfieri AA, Muller EE (2016) Prevalence and herd-level risk factors for bovine tuberculosis in the State of Parana, Brazil. Semin Cienc Agrar 37:3611-3623

442. Sintayehu DW, Prins HHT, Heitkonig IMA, de Boer WF (2017) Disease transmission in animal transfer networks. Prev Vet Med 137:36-42

443. Smith CM, Downs SH, Mitchell A, Hayward AC, Fry H, Le Comber SC (2015) Spatial targeting for bovine tuberculosis control: can the locations of infected cattle be used to find infected badgers? PLoS One 10:14

444. Smith GC, Budgey R (2021) Simulating the next steps in badger control for bovine tuberculosis in England. PLoS One 16:8

445. Smith GC, Cheeseman CL (2002) A mathematical model for the control of diseases in wildlife populations: culling, vaccination and fertility control. Ecol Modell 150:45-53

446. Smith GC, Cheeseman CL, Clifton-Hadley RS (1997) Modelling the control of bovine tuberculosis in badgers in England: culling and the release of lactating females. J Appl Ecol 34:1375-1386

447. Smith GC, Cheeseman CL, Clifton-Hadley RS, Wilkinson D (2001) A model of bovine tuberculosis in the badger *Meles meles*: an evaluation of control strategies. J Appl Ecol 38:509-519

448. Smith GC, Cheeseman CL, Wilkinson D, Clifton-Hadley RS (2001) A model of bovine tuberculosis in the badger *Meles meles*: the inclusion of cattle and the use of a live test. J Appl Ecol 38:520-535

449. Smith GC, Delahay RJ, McDonald RA, Budgey R (2016) Model of selective and non-selective management of badgers (*Meles meles*) to control bovine tuberculosis in badgers and cattle. PLoS One 11:16

450. Smith GC, McDonald RA, Wilkinson D (2012) Comparing badger (*Meles meles*) management strategies for reducing tuberculosis incidence in cattle. PLoS One 7:11

451. Smith GC, Richards MS, CliftonHadley RS, Cheeseman CL (1995) Modelling bovine tuberculosis in badgers in England: Preliminary results. Mammalia 59:639-650

452. Smith LA, Marion G, Swain DL, White PCL, Hutchings MR (2009) Inter- and intra-specific exposure to parasites and pathogens via the faecal-oral route: a consequence of behaviour in a patchy environment. Epidemiol Infect 137:630-643

453. Smith LA, White PCL, Marion G, Hutchings MR (2009) Livestock grazing behavior and inter- versus intraspecific disease risk via the fecal-oral route. Behav Ecol 20:426-432

454. Srinivasan S, Easterling L, Rimal B, Niu XM, Conlan AJK, Dudas P, Kapur V (2018) Prevalence of bovine tuberculosis in India: A systematic review and meta-analysis. Transbound Emerg Dis 65:1627-1640

455. Stanski K, Lycett S, Porphyre T, Bronsvoort BMD (2021) Using machine learning improves predictions of herd-level bovine tuberculosis breakdowns in Great Britain. Sci Rep 11:10

456. Swift BMC, Barron ES, Christley R, Corbetta D, Grau-Roma L, Jewell C, O'Cathail C, Mitchell A, Phoenix J, Prosser A, Rees C, Sorley M, Verin R, Bennett M (2021) Tuberculosis in badgers where the bovine tuberculosis epidemic is expanding in cattle in England. Sci Rep 11:10

457. Swinton J, Tuyttens F, Macdonald D, Nokes DJ, Cheeseman CL, CliftonHadley R (1997) Comparison of fertility control and lethal control of bovine tuberculosis in badgers: The impact of perturbation induced transmission. Philos Trans R Soc B 352:619-631

458. Tanner E, White A, Acevedo P, Balseiro A, Marcos J, Gortazar C (2019) Wolves contribute to disease control in a multi-host system. Sci Rep 9:12

459. Tanner E, White A, Lurz PWW, Gortazar C, Diez-Delgado I, Boots M (2019) The critical role of infectious disease in compensatory population growth in response to culling. Am Nat 194:e1-e12

460. Tasmi, D. Aldila, E. Soewono, N. Nuraini (2016) Mathematical model for transmission of tuberculosis in badger population with vaccination. AIP Conf Proc 1723:030021

461. Thompson AK, Samuel MD, Van Deelen TR (2008) Alternative feeding strategies and potential disease transmission in Wisconsin white-tailed deer. J Wildl Manag 72:416-421

462. Tipu MY, Chaudhary ZI, Younus M, Rabbani M (2012) A cross sectional study of *Mycobacterium bovis* in dairy cattle in and around Lahore City, Pakistan. Pakistan J Zool 44:393-398

463. Tolhurst BA, Delahay RJ, Walker NJ, Ward AI, Roper TJ (2009) Behaviour of badgers (*Meles meles*) in farm buildings: Opportunities for the transmission of *Mycobacterium bovis* to cattle? Appl Anim Behav Sci 117:103-113

464. Tolhurst BA, Ward AI, Delahay RJ, MacMaster AM, Roper TJ (2008) The behavioural responses of badgers (*Meles meles*) to exclusion from farm buildings using an electric fence. Appl Anim Behav Sci 113:224-235

465. Tomlinson AJ, Chambers MA, Carter SP, Wilson GJ, Smith GC, McDonald RA, Delahay RJ (2013) Heterogeneity in the risk of *Mycobacterium bovis* infection in European badger (*Meles meles*) cubs. Epidemiol Infect 141:1458-1466

466. Tompkins DM, Ramsey DSL, Cross ML, Aldwell FE, de Lisle GW, Buddle BM (2009) Oral vaccination reduces the incidence of tuberculosis in free-living brushtail possums. Proc Biol Sci 276:2987-2995

467. Tosa MI, Schauber EM, Nielsen CK (2017) Localized removal affects white-tailed deer space use and contacts. J Wildl Manag 81:26-37

468. Tratalos JA, Madden JM, McGrath G, Graham DA, Collins AB, More SJ (2020) Spatial and network characteristics of Irish cattle movements. Prev Vet Med 183:17

469. Trewby H, Wright D, Breadon EL, Lycett SJ, Mallon TR, McCormick C, Johnson P, Orton RJ, Allen AR, Galbraith J, Herzyk P, Skuce RA, Biek R, Kao RR (2016) Use of bacterial whole-genome sequencing to investigate local persistence and spread in bovine tuberculosis. Epidemics 14:26-35

470. Triguero-Ocana R, Laguna E, Jimenez-Ruiz S, Fernandez-Lopez J, Garcia-Bocanegra I, Barasona JA, Risalde MA, Montoro V, Vicente J, Acevedo P (2021) The wildlife-livestock interface on extensive free-ranging pig farms in central Spain during the "montanera" period. Transbound Emerg Dis 68:2066-2078

471. Triguero-Ocana R, Martinez-Lopez B, Vicente J, Barasona JA, Martinez-Guijosa J, Acevedo P (2020) Dynamic network of interactions in the wildlife-livestock interface in mediterranean Spain: An epidemiological point of view. Pathogens 9:16

472. Tschopp R, Bekele S, Moti T, Young D, Aseffa A (2015) Brucellosis and bovine tuberculosis prevalence in livestock from pastoralist communities adjacent to Awash National Park, Ethiopia. Prev Vet Med 120:187-194

473. Tschopp R, Schelling E, Hattendorf J, Aseffa A, Zinsstag J (2009) Risk factors of bovine tuberculosis in cattle in rural livestock production systems of Ethiopia. Prev Vet Med 89:205-211

474. Tschopp R, Schelling E, Hattendorf J, Young D, Aseffa A, Zinsstag J (2010) Repeated cross-sectional skin testing for bovine tuberculosis in cattle kept in a traditional husbandry system in Ethiopia. Vet Rec 167:250-256

475. Tsegaye W, Aseffa A, Mache A, Mengistu Y, Stefan B, Ameni G (2010) Conventional and molecular epidemiology of bovine tuberculosis in dairy farms in Addis Ababa City, the capital of Ethiopia. IJARVM 8:143-151

476. Tulu B, Zewede A, Belay M, Zeleke M, Girma M, Tegegn M, Ibrahim F, Jolliffe DA, Abebe M, Balcha TT, Gumi B, Martineau HM, Martineau AR, Ameni G (2021) Epidemiology of bovine tuberculosis and its zoonotic implication in Addis Ababa Milkshed, Central Ethiopia. Front Vet Sci 8:9

477. Turgenbayev KA, Borsynbayeva AM, Plazun AA, Turgenbayev RK (2021) Tuberculosis prevalence in animals and humans in the Republic of Kazakhstan. Vet World 14:2362-2370

478. Tuyttens FAM, Delahay RJ, MacDonald DW, Cheeseman CL, Long B, Donnelly CA (2000) Spatial perturbation caused by a badger (*Meles meles*) culling operation: implications for the function of territoriality and the control of bovine tuberculosis (*Mycobacterium bovis*). J Anim Ecol 69:815-828

479. Ullah A, Khattak US, Ayaz S, Qureshi MS, Khan I, Jan IU, Khattak I, Taj R, Nigar S, Khan NU, Jan IU, Khattak I, Taj R, Nigar S, Khan NU, Khan MA, Sohail ML (2019) Bovine Tuberculosis (bTB): Prevalence and associated risk factors in large ruminants in the central zone of Khyber Pakhtunkhwa, Pakistan. Pakistan J Zool 51:127-133

480. van den Driessche P, Wang L, Zou XF (2007) Modeling diseases with latency and relapse. MBE 4:205-219

481. van Tonder AJ, Thornton MJ, Conlan AJK, Jolley KA, Goolding L, Mitchell AP, Dale J, Palkopoulou E, Hogarth PJ, Hewinson RG, Wood JLN, Parkhill J (2021) Inferring *Mycobacterium bovis* transmission between cattle and badgers using isolates from the Randomised Badger Culling Trial. PLoS Pathog 17:24

482. Vander Wal E, Edye I, Paquet PC, Coltman DW, Bayne E, Brook RK, Andres JA (2013) Juxtaposition between host population structures: implications for disease transmission in a sympatric cervid community. Evol Appl 6:1001-1011

483. Vander Wal E, Paquet PC, Andres JA (2012) Influence of landscape and social interactions on transmission of disease in a social cervid. Mol Ecol 21:1271-1282

484. VanderWaal KL, Picasso C, Enns EA, Craft ME, Alvarez J, Fernandez F, Gil A, Perez A, Wells S (2016) Network analysis of cattle movements in Uruguay: Quantifying heterogeneity for risk-based disease surveillance and control. Prev Vet Med 123:12-22

485. Varela-Castro L, Alvarez V, Sevilla IA, Barral M (2020) Risk factors associated to a high *Mycobacterium tuberculosis complex* seroprevalence in wild boar (*Sus scrofa*) from a low bovine tuberculosis prevalence area. PLoS One 15:15

486. Varela-Castro L, Gerrikagoitia X, Alvarez V, Geijo MV, Barral M, Sevilla IA (2021) A long-term survey on *Mycobacterium tuberculosis complex* in wild mammals from a bovine tuberculosis low prevalence area. Eur J Wildl Res 67:8

487. Varela-Castro L, Sevilla IA, Payne A, Gilot-Fromont E, Barral M (2021) Interaction Patterns between Wildlife and Cattle Reveal Opportunities for Mycobacteria Transmission in Farms from North-Eastern Atlantic Iberian Peninsula. Animals 11:20

488. Vazquez CB, Barral TD, Romero B, Queipo M, Merediz I, Quiros P, Armenteros JA, Juste R, Dominguez L, Dominguez M, Casais R, Balseiro A (2021) Spatial and temporal distribution of *Mycobacterium tuberculosis complex* infection in eurasian badger (*Meles meles*) and cattle in Asturias, Spain. Animals 11:14

489. Veloso FP, Baumgarten KD, Mota A, Ferreira F, Neto JSF, Grisi JHH, Dias RA, Amaku M, Telles EO, Goncalves VSP (2016) Prevalence and herd-level risk factors of bovine tuberculosis in the State of Santa Catarina. Semin Cienc Agrar 37:3659-3672

490. Vial F, Donnelly CA (2012) Localized reactive badger culling increases risk of bovine tuberculosis in nearby cattle herds. Biol Lett 8:50-53

491. Vial F, Johnston WT, Donnelly CA (2011) Local cattle and badger populations affect the risk of confirmed tuberculosis in british cattle herds. PLoS One 6:9

492. Vicente J, Barasona JA, Acevedo P, Ruiz-Fons JF, Boadella M, Diez-Delgado I, Beltran-Beck B, Gonzalez-Barrio D, Queiros J, Montoro V, de la Fuente J, Gortazar C (2013) Temporal trend of tuberculosis in wild ungulates from mediterranean Spain. Transbound Emerg Dis 60:92-103

493. Vicente J, Delahay RJ, Walker NJ, Cheeseman CL (2007) Social organization and movement influence the incidence of bovine tuberculosis in an undisturbed high-density badger *Meles meles* population. J Anim Ecol 76:348-360

494. Vicente J, Hofle U, Garrido JM, Fernandez-de-Maria IG, Acevedo P, Juste R, Barral M, Gortazar C (2007) Risk factors associated with the prevalence of tuberculosis-like lesions in fenced wild boar and red deer in south central Spain. BMC Vet Res 38:451-464

495. Vicente J, Hofle U, Garrido JM, Fernandez-de-Mera IG, Juste R, Barral M, Gortazar C (2006) Wild boar and red deer display high prevalences of tuberculosis-like lesions in Spain. BMC Vet Res 37:107-119

496. Vidondo B, Voelkl B (2018) Dynamic network measures reveal the impact of cattle markets and alpine summering on the risk of epidemic outbreaks in the Swiss cattle population. BMC Vet Res 14:11

497. Vinueza RL, Durand B, Zanella G (2022) Network analysis of cattle movements in Ecuador. Prev Vet Med 201:10

498. Volkova VV, Howey R, Savill NJ, Woolhouse MEJ (2010) Potential for transmission of infections in networks of cattle farms. Epidemics 2:116-122

499. Walter WD, Smith R, Vanderklok M, VerCauteren KC (2014) Linking bovine tuberculosis on cattle farms to white-tailed deer and environmental variables using bayesian hierarchical analysis. PLoS One 9:8

500. Ward AI, Smith GC, Etherington TR, Delahay RJ (2009) Estimating the risk of cattle exposure to tuberculosis posed by wild deer relative to badgers in England and Wales. J Wildl Dis 45:1104-1120

501. Weber N, Bearhop S, Dall SRX, Delahay RJ, McDonald RA, Carter SP (2013) Denning behaviour of the European badger (*Meles meles*) correlates with bovine tuberculosis infection status. Behav Ecol Sociobiol 67:471-479

502. Weinhaupl I, Schopf KC, Khaschabi D, Kapaga AM, Msami HM (2000) Investigations on the prevalence of bovine tuberculosis and brucellosis in dairy cattle in Dar es Salaam region and in zebu cattle in Lugoba area, Tanzania. Trop Anim Health Prod 32:147-154

503. White PCL, Benhin JKA (2004) Factors influencing the incidence and scale of bovine tuberculosis in cattle in southwest England. Prev Vet Med 63:1-7

504. White PCL, Harris S (1995) Bovine tuberculosis in badger (*Meles meles*) populations in southwest england - an assessment of past, present and possible future control strategies using simulation modeling. Philos Trans R Soc B 349:415-432

505. White PCL, Harris S (1995) Bovine tuberculosis in badger (*Meles meles*) populations in southwest england - the use of a spatial stochastic simulation-model to understand the dynamics of the disease. Philos Trans R Soc B 349:391-413

506. White PCL, Lewis AJG, Harris S (1997) Fertility control as a means of controlling bovine tuberculosis in badger (*Meles meles*) populations in south-west England: predictions from a spatial stochastic simulation model. Proc Biol Sci 264:1737-1747

507. White PW, Martin SW, De Jong MCM, O'Keeffe JJ, More SJ, Frankena K (2013) The importance of 'neighbourhood' in the persistence of bovine tuberculosis in Irish cattle herds. Prev Vet Med 110:346-355

508. Whyte BI, Ross JG, Buckley HL (2014) Changes in Australian brushtail possum (*Trichosurus vulpecula*) den site use following density reduction. New Zealand J Ecol 38:147-151

509. Wielgus E, Caron A, Bennitt E, De Garine-Wichatitsky M, Cain B, Fritz H, Miguel E, Cornelis D, Chamaille-Jammes S (2021) Inter-group social behavior, contact patterns and risk for pathogen transmission in cape buffalo populations. J Wildl Manag 85:1574-1590

510. Wilber MQ, Pepin KM, Campa H, Hygnstrom SE, Lavelle MJ, Xifara T, VerCauteren KC, Webb CT (2019) Modelling multi-species and multi-mode contact networks: Implications for persistence of bovine tuberculosis at the wildlife-livestock interface. J Appl Ecol 56:1471-1481

511. Wilkinson D, Bennett R, McFarlane I, Rushton S, Shirley M, Smith GC (2009) Cost-benefit analysis model of badger (*Meles meles*) culling to reduce cattle herd tuberculosis breakdowns in Britain, with particular reference to badger perturbation. J Wildl Dis 45:1062-1088

512. Wilkinson D, Smith GC, Delahay RJ, Cheeseman CL (2004) A model of bovine tuberculosis in the badger *Meles meles*: an evaluation of different vaccination strategies. J Appl Ecol 41:492-501

513. Winkler B, Mathews F (2015) Environmental risk factors associated with bovine tuberculosis among cattle in high-risk areas. Biol Lett 11:5

514. Wint GRW, Robinson TP, Bourn DM, Durr PA, Hay SI, Randolph SE, Rogers DJ (2002) Mapping bovine tuberculosis in Great Britain using environmental data. Trends Microbiol 10:441-444

515. Wolfe DM, Berke O, Kelton DF, White PW, More SJ, O'Keeffe J, Martin SW (2010) From explanation to prediction: A model for recurrent bovine tuberculosis in Irish cattle herds. Prev Vet Med 94:170-177

516. Woodroffe R, Donnelly CA, Chapman K, Ham C, Moyes K, Stratton NG, Cartwright SJ (2021) Successive use of shared space by badgers and cattle: implications for *Mycobacterium bovis* transmission. J Zool 314:132-142

517. Woodroffe R, Donnelly CA, Cox DR, Bourne FJ, Cheeseman CL, Delahay RJ, Gettinby G, McInerney JP, Morrison WI (2006) Effects of culling on badger *Meles meles* spatial organization: implications for the control of bovine tuberculosis. J Appl Ecol 43:1-10

518. Woodroffe R, Donnelly CA, Cox DR, Gilks P, Jenkins HE, Johnston WT, Le Fevre AM, Gourne FJ, Cheeseman CL, Clifton-Hadley RS, Gettinby G, Hewinson RG, McInerney JP, Mitchell AP, Morrison WI, Watkins GH (2009) Bovine tuberculosis in cattle and badgers in localized culling areas. J Wildl Dis 45:128-143

519. Woodroffe R, Donnelly CA, Ham C, Jackson SYB, Moyes K, Chapman K, Stratton NG, Cartwright SJ (2016) Badgers prefer cattle pasture but avoid cattle: implications for bovine tuberculosis control. Ecol Lett 19:1201-1208

520. Woodroffe R, Donnelly CA, Ham C, Jackson SYB, Moyes K, Chapman K, Stratton NG, Cartwright SJ (2017) Ranging behaviour of badgers *Meles meles* vaccinated with Bacillus Calmette Guerin. J Appl Ecol 54:718-725

521. Woodroffe R, Donnelly CA, Ham C, Jackson SYB, Moyes K, Chapman K, Stratton NG, Cartwright SJ (2017) Use of farm buildings by wild badgers: implications for the transmission of bovine tuberculosis. Eur J Wildl Res 63:9

522. Woodroffe R, Donnelly CA, Jenkins HE, Johnston WT, Cox DR, Bourne FJ, Cheeseman CL, Delahay RJ, Clifton-Hadley RS, Gettinby G, Gilks P, Hewinson RG, McInerney JP, Morrison WI (2006) Culling and cattle controls influence tuberculosis risk for badgers. PNAS USA 103:14713-14717

523. Woodroffe R, Donnelly CA, Johnston WT, Bourne FJ, Cheeseman CL, Clifton-Hadley RS, Cox DR, Gettinby G, Hewinson RG, Le Fevre AM, Mcinerney JP, Morrison WI (2005) Spatial association of *Mycobacterium bovis* infection in cattle and badgers Meles meles. J Appl Ecol 42:852-862

524. Woodroffe R, Donnelly CA, Wei G, Cox DR, Bourne FJ, Burke T, Butlin RK, Cheeseman CL, Gettinby G, Gilks P, Hedges S, Jenkins HE, Johnston WT, McInerney JP, Morrison WI, Pope LC (2009) Social group size affects *Mycobacterium bovis* infection in European badgers (*Meles meles*). J Anim Ecol 78:818-827

525. Woodroffe R, Frost SDW, Clifton-Hadley RS (1999) Attempts to control tuberculosis in cattle by removing infected badgers: constraints imposed by live test sensitivity. J Appl Ecol 36:494-501

526. Woodroffe R, Gilks P, Johnston WT, Le Fevre AM, Cox DR, Donnelly CA, Bourne FJ, Cheeseman CL, Gettinby G, McInerney JP, Morrison WI (2008) Effects of culling on badger abundance: implications for tuberculosis control. J Zool 274:28-37

527. Wright DM, Reid N, Montgomery WI, Allen AR, Skuce RA, Kao RR (2015) Herd-level bovine tuberculosis risk factors: assessing the role of low-level badger population disturbance. Sci Rep 5:11

528. Xu F, Tian LL, Li Y, Zhang XL, Qi YY, Jing ZG, Pan YY, Zhang L, Fan XX, Wang M, Zeng Q, Fan W (2021) High prevalence of extrapulmonary tuberculosis in dairy farms: Evidence for possible gastrointestinal transmission. PLoS One 16:12

529. Zanella G, Bar-Hen A, Boschiroli ML, Hars J, Moutou F, Garin-Bastuji B, Durand B (2012) Modelling transmission of bovine tuberculosis in red deer and wild boar in Normandy, France. ZPHOAH 59:170-178

530. Zanella G, Durand B, Hars J, Moutou F, Garin-Bastuji B, Duvauchelle A, Ferme M, Karoul C, Boschiroli ML (2008) *Mycobacterium bovis* in wildlife in France. J Wildl Dis 44:99-108

531. Zendejas-Martinez H, Peterson AT, Milian-Suazo F (2008) Coarse-scale spatial and ecological analysis of tuberculosis in cattle: an investigation in Jalisco, Mexico. Geospat Health 3:29-38

532. Zhu XJ, Yan Y, Wang ZJ, Zhang KL, Chen YY, Peng YC, Peng QJ, Guo AZ, Robertson ID, Aleri J (2021) An abattoir-based study on the prevalence of bovine tuberculosis from culled adult dairy cows in Wuhan, China. Prev Vet Med 196:9
